# BC4707 Is a Major Facilitator Superfamily Multidrug Resistance Transport Protein from *Bacillus cereus* Implicated in Fluoroquinolone Tolerance

**DOI:** 10.1371/journal.pone.0036720

**Published:** 2012-05-16

**Authors:** Roger Simm, Aniko Vörös, Jaakko V. Ekman, Marianne Sødring, Ingerid Nes, Jasmin K. Kroeger, Massoud Saidijam, Kim E. Bettaney, Peter J. F. Henderson, Mirja Salkinoja-Salonen, Anne-Brit Kolstø

**Affiliations:** 1 Laboratory for Microbial Dynamics, Department of Pharmaceutical Biosciences, School of Pharmacy, University of Oslo, Oslo, Norway; 2 Department of Biosciences, Biocenter 1, University of Helsinki, Helsinki, Finland; 3 Astbury Centre for Structural Molecular Biology, Institute of Membrane and Systems Biology, University of Leeds, Leeds, United Kingdom; 4 School of Medicine, Hamedan University of Medical Sciences, Hamedan, Iran; University of Cambridge, United Kingdom

## Abstract

Transcriptional profiling highlighted a subset of genes encoding putative multidrug transporters in the pathogen Bacillus cereus that were up-regulated during stress produced by bile salts. One of these multidrug transporters (BC4707) was selected for investigation. Functional characterization of the BC4707 protein in Escherichia coli revealed a role in the energized efflux of xenobiotics. Phenotypic analyses after inactivation of the gene bc4707 in Bacillus cereus ATCC14579 suggested a more specific, but modest role in the efflux of norfloxacin. In addition to this, transcriptional analyses showed that BC4707 is also expressed during growth of B. cereus under non-stressful conditions where it may have a role in the normal physiology of the bacteria. Altogether, the results indicate that bc4707, which is part of the core genome of the B. cereus group of bacteria, encodes a multidrug resistance efflux protein that is likely involved in maintaining intracellular homeostasis during growth of the bacteria.

## Introduction

*Bacillus cereus sensu lato* (the *Bacillus cereus* group of bacteria) are Gram-positive rod-shaped bacteria that readily form endospores under unfavorable growth conditions. They comprise six phenotypically diverse, but genetically closely related, species - *B. cereus sensu stricto*, *B. anthracis*, *B. thuringiensis*, *B. weihenstephaniensis*, *B mycoides* and *B. pseudomycoides* [1]. Among these bacteria, the last three species are generally considered non-pathogenic. *B. thuringiensis* is an insect pathogen commercially used as a biopesticide. *B. anthracis* is notorious for being the agent causing anthrax in humans and animals [2] as well as for its potential role in biological warfare including bioterrorism [3]. *B. cereus sensu stricto* (from now on referred to as *B. cereus*) is primarily associated with food borne, self-limiting gastrointestinal infections. It is also an opportunistic pathogen that causes local infections following trauma as well as systemic diseases in predisposed patients [4]–[6]. Although *B. cereus* is commonly reported as a soil bacterium, the life style is not completely understood. Spores have been isolated from different types of soil [7] as well as faeces of healthy humans [8], [9]. There are reports of *B. cereus* found in the rhizosphere of plants [10] and guts of invertebrates [11]. The current view is that soil-dwelling spores germinate and grow, either in an animal host or in the rhizosphere of plants, suggesting that the natural lifestyle of *B. cereus* often involves symbiotic or pathogenic interactions with animals and plants. The diverse environments where *B. cereus* is found suggest that these bacteria survive and grow in competition with other microorganisms as well as in a range of challenging conditions such as different temperatures, extremes of pH and toxic compounds.

In order to survive challenging extracellular conditions, it is essential to maintain homeostasis in the intracellular environment. This is partly achieved through the action of different types of membrane proteins that transport nutrients and ions into the cell and excrete waste products and toxic compounds from the cell. According to the transportdb database [12], [13] the *B. cereus* type strain (ATCC14579) has 390 putative membrane transporters, including approximately 100 predicted multidrug transporters. The high number of predicted membrane transporters encountered in *B. cereus* species may well reflect the diverse environments where they are found.

Multidrug transporters are energy dependent membrane proteins that extrude a wide array of structurally and functionally dissimilar compounds. Multidrug transporters play a significant role in resistance of bacteria to antimicrobial compounds [14]–[16]. It has also been suggested that multidrug transporters have additional/alternative roles in the normal physiology of bacteria, facilitating survival in their ecological niche [17]–[19]. Multidrug transporters are categorized into five superfamilies [20] i. Major Facilitator Superfamily (MFS), ii. Resistance nodulation cell division (RND), iii. Small multidrug resistance (SMR), iv. Multidrug and toxic compound extrusion (MATE) and v. ATP-binding cassette (ABC). The ABC transporters use the energy from ATP hydrolysis directly to force drug expulsion. The other four families of multidrug transporters are secondary metabolite transporters driving drug efflux with either the proton or sodium gradients that are established across the cytoplasmic membrane of cells during respiration (or by ATP hydrolysis in strictly fermentative organisms). Although at present, most of the transporters encoded in *B. cereus* remain uncharacterized, it was shown that a set of membrane transporters, including several putative multidrug transporters were upregulated in *B. cereus* ATCC14579 when challenged with sub-lethal concentrations of bile salts [21]. In addition, a partly overlapping set of transporters was induced by sub-lethal concentration of acetic acid [22]. Bacteria would encounter similar stress during passage through the gastrointestinal tract, suggesting that these transporters could be part of a survival system expressed in bacteria in order to inhabit such hostile environments.

In this study, we initiated the biochemical and functional characterization of one putative multidrug transporter (BC4707) shown to be induced by sub-lethal concentrations of both bile salts and acetic acid; by cloning, expressing and characterizing its activities in an *E. coli* host. In parallel, we deleted the gene (*bc4707*) and examined its role in the *B. cereus* ATCC14579 under non-stressful conditions such as exponential growth in LB-medium as well as stressful conditions including bile salts stress, metabolic stress, antibiotics and low pH.

## Results

### BC4707 is not essential for *B. cereus* to survive bile salts stress

**Figure 1:**
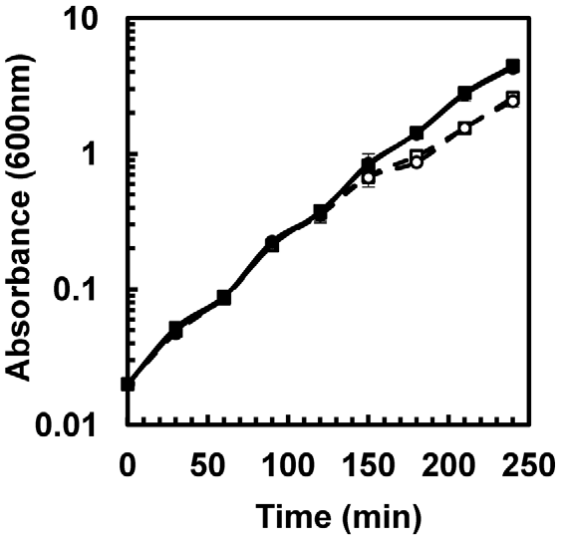
Comparison of the growth of *Bacillus cereus* ATCC14579 and its isogenic *Δbc4707* mutant. Growth was assayed for the wild type (circles) and the mutant (squares) by monitoring the absorbance at 600 nm in LB medium (solid lines; closed symbols) and during 50 µg/ml bile salts stress initiated at OD_600_ of 0.5 (dashed lines; open symbols). Shown are averages and standard deviations of two independent experiments.

Induction of *bc4707* transcription in *B. cereus* upon bile salt stress [21], suggested a role in bile salts efflux. To test the role of BC4707 in bile salts protection, a markerless *Δbc4707* mutant was constructed in *B. cereus* ATCC14579 and the two strains were compared under growth in LB medium as well as under bile salts stress. It was revealed that although growth of the bacteria was repressed in the prescence of 50 µg/ml bile salts the *Δbc4707* mutant and the wild type grow in a similar fashion (Fig. 1). This result indicated that although *bc4707* expression is induced under bile salts stress, the protein is not essential for bile salts protection.

### Transcriptional profiling suggests that BC4707 is not involved in protection of *B. cereus* against bile salts stress

Considering that *bc4707* expression was induced by bile salts stress whereas BC4707 was non-essential for growth in the presence of sub-lethal concentrations of bile salts, we hypothesized that a compensatory mechanism was induced in the *Δbc4707* mutant under such conditions. In order to test for compensatory mechanisms, a series of microarray analyses were performed comparing the transcriptional profiles of the wild type *B. cereus* and the *Δbc4707* mutant under bile salts stress. Bacteria were grown to an OD_600_ of 0.5. At this point bile salt was added to a concentration of 50 µg/ml and the cultures were incubated for 15 additional minutes. RNA was prepared for each strain at OD_600_ of 0.5 (WT^OD0.5^, mutant^OD0.5^) as well as after 15 min bile salts stress (WT^BS15^, mutant^BS15^). To determine the bile salt induced stress response in LB medium, the mRNA content of the bile salt stressed cells was compared to the mRNA content of the pre-stressed cells by microarray analysis (i.e. WT^BS15^ was compared to WT^OD0.5^ and mutant^BS15^ was compared to mutant^OD0.5^. Arrayexpress accession numbers E-MEXP-3535 and E-MEXP-3536 respectively). These microarray analyses demonstrated a substantial stress response in both the wild type and the *Δbc4707* mutant (Table S1), with significant differential regulation of approximately 10% of the genome, including at least 10 efflux proteins (counting *bc4707* in the wt strain, see also Kristoffersen *et al.* [21]). The transcriptional profiles of the wild type and mutant after 15 min bile salts stress were similar (Table S1) with few and negligible differences between the two data sets. To identify potential compensatory mechanisms that allow the *Δbc4707* mutant to grow similar to the wild type under bile salt stress, the mRNA content of the *Δbc4707* strain after bile salts stress for 15 min (mutant^BS15^) was compared to the corresponding mRNA of the wild type (WT^BS15^). This microarray analysis (Arrayexpress accession number E-MEXP-3529) confirmed that there are few differences between the two strains in the bile salts induced stress response (Table 1). Four genes were differentially expressed more than 1.5-fold. Three of these genes were up-regulated in the *Δbc4707* mutant: *bc2989* annotated as a hypothetical protein; *bc4111*, which, from sequence similarity, is described as a bifunctional 3,4-dihydroxy-2-butanone 4-phosphate synthase/GTP cyclohydrase II protein, involved in riboflavin synthesis; and *bc4904*, annotated as a hydrolase of the alpha/beta fold family. Furthermore, the cold shock protein CspD (*bc4859*), was down regulated in the*Δbc4707* mutant compared to the wild type. This indicates that the absence of BC4707 does not induce a major compensatory response to bile salts stress at the mRNA level.

**Table 1:** Transcriptional profile of *B. cereus* ATCC14579 *Δbc4707* (AH1598) compared to wild type (AH1709) grown to logarithmic stage (OD_600_∼0.5) in rich medium followed by bile salt stress for 15 min.

Gene	Predicted protein function	*Δbc4707*/WT
		(fold change)
*bc2989*	Hypothetical protein	1.68
*bc4111*	Bifunctional 3,4-dihydroxy-2-butanone 4-phosphate synthase/GTP cyclohydrase II protein	1.51
*bc4707*	drug resistance transporter, EmrB/QacA family	**0.32**
*bc4859*	Cold shock protein CspD	0.61
*bc4904*	Hydrolase, alpha/beta fold family	1.96

### BC4707 is not involved in the acid stress defense of *B. cereus*

According to Mols et al. *bc4707* is slightly up-regulated under sub-lethal acetic acid stress [22], indicating a potential role for BC4707 in maintaining the intracellular pH of *B. cereus* in an acidic environment. To test this, the experiments described by Mols et al. were reproduced. Bacteria were grown in a neutrally buffered BHI medium to mid-logarithmic phase, at which point the medium was acidified and the OD was measured for an additional 4.5 hours. No difference was observed between the wild type and *Δbc4707* mutant (data not shown), indicating a non-essential role for BC4707 under acetic acid shock.

### Inactivation of the *bc4707* gene renders *B. cereus* more susceptible to norfloxacin

**Figure 2:**
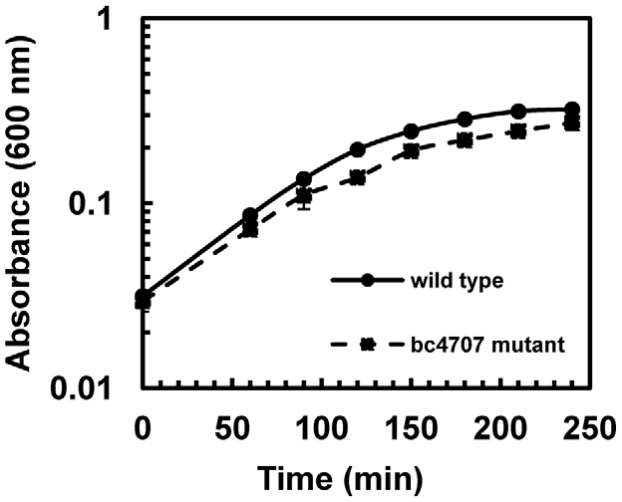
Growth of *B. cereus* ATCC14579 and *Δbc4707* mutant under stress with sublethal concentration of norfloxacin. Over night cultures were diluted 1∶100 and grown to mid-logarithmic phase. These cultures were diluted to OD_600_ of 0.02 in 25 ml LB and norfloxacin was added. The cultures were incubated for 240 min at 30°C and 220 rpm shaking. The growth curves show averages and standard deviations of two independent experiments.

To elucidate the role of BC4707 in *B. cereus* drug tolerance, the markerless knock out mutant was compared to the wild type in susceptibility assays (Table 2). The *Δbc4707* mutant and wild type strains only differed in the sensitivity to one (norfloxacin) of 12 toxic compounds tested. The two-fold difference in susceptibility to norfloxacin was consistent between the wild type and *Δbc4707* mutant. Growth curves of the wild type and mutant supplemented with a sub-lethal concentration of norfloxacin, demonstrated a moderate, but consistent reduction in growth of the *Δbc4707* mutant compared to the wild type (Fig. 2). Taken together, BC4707 is involved in norfloxacin tolerance of *B. cereus* ATCC14579.

**Table 2:** Susceptibilities of *B. cereus* ATCC14579 (wild type) compared to its isogenic *Δbc4707* mutant.

	MIC (µg/ml)		
	*B. cereus ATCC14579*		
Compound	Wild type	*Δbc4707*	§
Chloramphenicol	2.5	2.5	1
Ciprofloxacin	0.32	0.32	1
Bile salts	125	125	1
Deoxycholate	250	250	1
SDS	100	100	1
Crystal violet	0.1	0.1	1
Kanamycin	15	15	1
Nalidixic acid	1.875	1.875	1
Erythromycin	0.2	0.2	1
Ethidium bromide	40	40	1
Norfloxacin	1.25	0.625	2
Tetracycline	2.5	2.5	1

§ represents the fold difference in MIC between the wild type *B. cereus* ATCC14579 and its isogenic *Δbc4707* mutant.

### Microarray analysis suggests a physiological role for BC4707 in exponential growth of *B. cereus*

**Figure 3:**
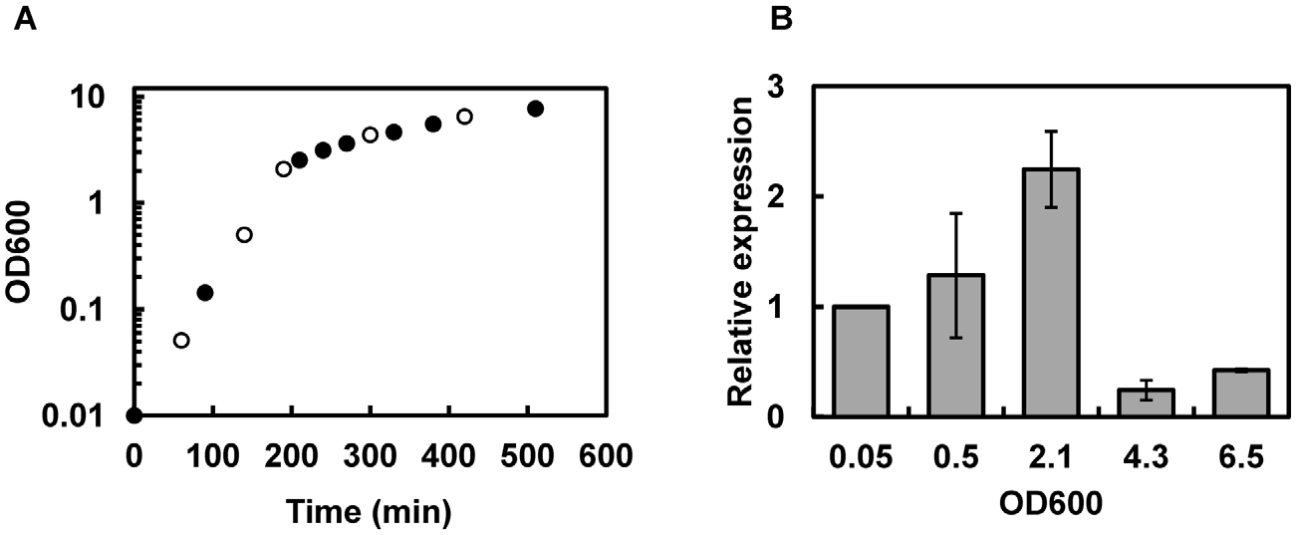
Growth of *B. cereus* ATCC14579 and expression analysis of *bc4707*. A. Bacteria were grown in LB-medium. Circles indicate OD_600_ measurements. Bacteria were harvested for RNA extraction (open circles). B. Gene expression analysis of *bc*4707 by qRT-PCR. Averages and standard deviations from three experiments are shown. The relative expression at different OD_600_ values is compared to the expression level at OD_600_ of 0.05. 16S RNA was used as a control to normalize the data.

**Figure 4:**
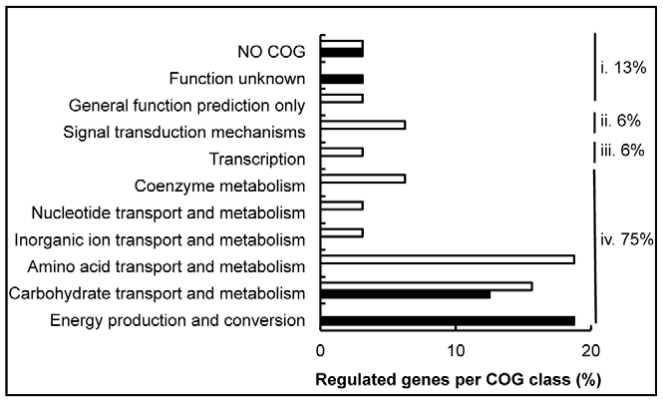
COG distribution of differentially regulated genes in exponentially growing *Δbc4707* mutant and wild type. The proportion of genes belonging to a COG class is shown as percentage of the total number of differentially regulated genes. Open bars indicate downregulated genes in the mutant compared to the wild type , solid bars represent upregulated genes. i. the proportion of regulated genes that are poorly characterized, ii. the proportion of genes with a predicted function in cellular processes and signaling, iii. the proportion of genes involved in information storage and processing, iv. the proportion of regulated genes involved in metabolism and transport.

Although bioinformatics analysis has predicted BC4707 to be a multidrug transporter, susceptibility testing of the *Δbc4707* mutant only identified norfloxacin as a potential substrate (Table 2). A few multidrug transporters have been shown to have physiological functions in addition to multidrug efflux [17], [18], [23]–[25]. The large number of putative multidrug transporters in *B. cereus* suggests alternative or additional functions to multidrug efflux for some of these transporters, possibly under conditions that do not impose exogenous stress on the bacteria. We therefore measured the transcription level of *bc4707* under logarithmic growth in rich medium. Quantitative RT-PCR analysis at different time points of *B. cereus* ATCC14579 growing in LB medium indicated that although the absolute expression level of *bc4707* is relatively low (data not shown) it is increasingly expressed during logarithmic phase up to the onset of the transition preceding stationary phase in *B. cereus* (Fig. 3). The increasing expression of *bc4707* during logarithmic phase supported the hypothesis of a potential role for BC4707 under exogenously imposed non-stressful growth conditions. Growth of the *Δbc4707* mutant in LB-medium, however, was identical to the growth of the wild type strain (Fig. 1), suggesting a non-essential role for BC4707 in rich medium. Potentially, overlapping functions of constitutively expressed efflux proteins may conceal the function of the moderately expressed BC4707. Alternatively, a compensatory mechanism may be induced that masks the necessity for the BC4707 efflux protein. Microarray analysis was therefore carried out in mid logarithmic phase at OD_600_ 0.5 of cells growing in LB medium (WT^OD0.5^, mutant^OD0.5^), comparing the wild type and the BC4707 deletion mutant (Arrayexpress accession number E-MEXP-3537). The results (Fig. 4 and Table 3) revealed that although the *Δbc4707* mutant and the wild type displayed the same growth rate, 27 genes were differentially regulated, at least 1.5 fold at OD_600_ 0.5. Thirteen genes were up-regulated, whereas 14 genes were down-regulated, thus indicating a potential role for BC4707 in the normal physiology of *B. cereus* ATCC14579 under exogenously non-stressful conditions, most likely by relieving the cell of toxic metabolites.

**Table 3:** Transcriptional profile of *B. cereus* ATCC14579 *Δbc4707* (AH1598) compared to wild type (AH1709) under logarithmic growth (OD_600_∼0.5) in rich medium.

Gene	Predicted protein function	Δ*bc4707*/WT
		(fold change)
*bc0579*	malate sodium symporter	**2.06**
*bc0580*	malate dehydrogenase	1.61
*bc0659*	ribose operon repressor	1.54
*bc0660*	ribokinase	1.52
*bc0662*	ribose ABC transporter, ATP-binding protein	1.52
*bc0872*	cystine-binding protein	**0.41**
*bc0873*	cystine permease protein	**0.39**
*bc0874*	cystine ATP-binding protein	**0.44**
*bc0909*	oligopeptide ABC transporter, permease protein	0.63
*bc0942*	hypothetical protein	0.65
*bc1034*	glycerol uptake facilitator protein	**2.12**
*bc1035*	glycerol kinase	1.53
*bc1251*	dihydrolipoamide acetyltransferase	1.59
*bc1252*	2-oxoglutarate dehydrogenase, E1 component	1.57
*bc1739*	proton/sodium-glutamate symporter	1.65
*bc2045*	hypothetical protein	1.68
*bc2300*	oxalate∶formate antiporter, putative	1.57
*bc3718*	PTS system, fructose-specific IIABC component	0.60
*bc3719*	1-phosphofructokinase	0.60
*bc3720*	transcriptional regulator, DeoR family	**0.41**
*bc3760*	6-phospho-beta-glucosidase	0.59
*bc4242*	proton/sodium-glutamate symporter	**0.47**
*bc4366*	cystathionine beta-lyase	0.52
*bc4367*	cystein synthase A	**0.50**
*bc4368*	5′-methylthioadenosine/S-adenosylhomocysteine nucleosidase	0.56
*bc4369*	Dimethyladenosine transferase	**0.47**
*bc4396*	molybdopterin biosynthesis protein	0.64
*bc4707*	drug resistance transporter, EmrB/QacA family	**0.38**
*bc4789*	S-ribosylhomocysteinase	0.65
*bc5239*	enterotoxin/cell wall binding protein	1.80

Bold text indicates that the gene is differentially regulated more than or equal to two times (i.e. the values are ≥2 or ≤0.5).

### Phenotype analyses did not identify a condition where BC4707 is essential

To screen for potential substrates or conditions where BC4707 is essential, or has a major role, Phenotype MicroArray™ experiments were conducted. In these experiments, the metabolic activity was compared between the *Δbc4707* mutant and the wild type strain in 672 different conditions, including peptide nitrogen sources, carbon sources, and pH. There were no consistent differences in metabolic activity between the wild type and mutant under any of the conditions (PM plates 1, 2, 6–8 and 10) testing for carbon source, peptide nitrogen source or pH preferences (data not shown).

To complement the phenotype microarray, growth tests were conducted in minimal medium using different compounds as sole carbon and/or nitrogen sources. The growth experiments were initially carried out in liquid cultures, but the OD_600_ measurements failed to give a reading, since bacteria exclusively grew as aggregates on the glass at the air-liquid interface. To circumvent this problem, subsequent experiments were carried out on GGGS-agar plates. In agreement with the phenotype microarrays, no consistent differences were found between the *Δbc4707* mutant and the wild type in growth behaviour (data not shown). There were indications of a small difference in motility behavior on GGGS plates, where the mutant appeared to be more motile than the wild type. However, a consistent difference between the wild type and mutant could not be confirmed on swarming or swimming plates (data not shown).

### Expression of the *bc4707* gene in *Escherichia coli*

Although *bc4707* expression is induced under bile salts stress [21] as well as under acetic acid shock [22], the protein is not essential for growth under such conditions (Fig. 1, Table 1 and data not shown). Sequence similarity has classified BC4707 as a multidrug transporter of the Major Facilitator Superfamily [12]. Deletion of *bc4707* from *B. cereus* ATCC14579 only affected growth of the mutant in one of 12 tested toxic compounds. It is likely that redundancy among efflux proteins conceal an eventual multidrug transport ability of BC4707. A common and sensitive method to demonstrate multidrug transport capacity in a membrane protein is by testing for increased drug tolerance after heterologues expression in an *E. coli* strain that is hypersensitive to multiple drugs. For this purpose, the gene (*bc4707*) was cloned into the *E. coli* expression vector pTTQ18 as described previously [26]. The resulting plasmid potentiates IPTG inducible heterologous expression of a BC4707-RGSHis_6_ tag fusion protein in *E. coli*.

**Figure 5:**
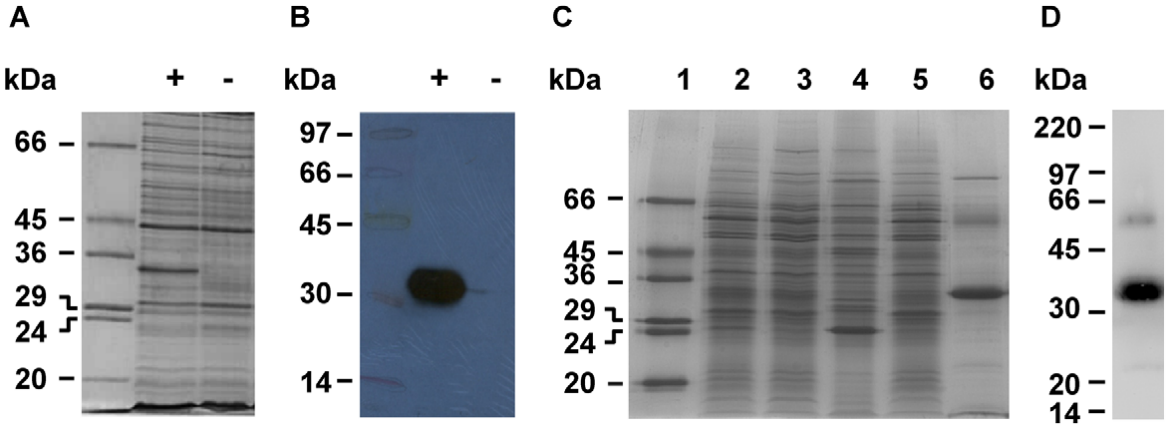
Expression and purification of BC4707-RGSHis_6_. Protein expression was induced for 3 hours by addition of IPTG (1 mM) to exponentially growing bacteria. Cells were harvested and mixed membrane preparations were made from each culture. The proteins in the mixed membrane fractions prepared by the water-lysis technique [47] were separated by SDS-PAGE and detected by: (A) Coomassie Blue stain; or (B) Western blotting with an antibody to the RGSHis_6_-tag (+ and − represent mixed membrane preparations of induced and uninduced cells, respectively). Purification of BC4707-RGSHis_6_ was done by immobilized metal affinity chromatography (IMAC). Staining with Coomassie Blue (C) shows the enrichment and purity of BC4707-RGSHis_6_ at different stages of purification. The gel was loaded as follows: 1) molecular weight marker 2) inner membranes solubilized in dodecyl-beta-D-maltoside detergent; 3) supernatant of the solubilized inner membranes after ultracentrifugation; 4) pellet of the solubilized inner membrane fraction after ultracentrifugation; 5) flow-through after IMAC-binding; 6) elution fraction (5 µg protein). Western blot (D) of the final elution fraction (7.5 µg protein). The sizes of the bands in the molecular weight markers are indicated.

Induction of protein expression with IPTG yielded a relatively high level of BC4707-RGSHis_6_ in the membranes of the expression host BL21 (DE3), as demonstrated by Coomassie Blue staining as well as Western blotting using an antibody raised against the RGSHis_6_ tag (Fig. 5 a and b). The observed molecular weight of the BC4707-RGSHis_6_ protein after SDS-PAGE (Fig. 5) was substantially lower (35 kDa) than that (52.5 kDa) predicted from the amino acid sequence, a common feature for membrane proteins [27], [28]. The protein was solubilized from the inner membrane of the *E. coli* host using the detergent dodecyl–beta-D-maltoside (DDM) and purified to approximately 90% homogeneity by immobilized metal affinity chromatography (IMAC) as described in Materials and Methods (Fig. 5 c). The impurities detected by Coomassie staining in the purified protein fraction, likely represent histidine-containing host proteins that co-purified with the target protein [29]. Two protein bands in addition to the target one of 35 kDa repeatedly showed up on Western blots (Fig. 5 d). The band of higher molecular weight than the target, probably represented the completely unfolded form of BC4707-RGSHis_6_ [27], whereas the faint band of lower molecular weight is likely a degradation product of the target protein. Edman degradation confirmed that the N-terminal amino acid sequence of the protein that was detected as the major band on the Coomassie stained gel was the *B. cereus* BC4707 protein (data not shown).

**Figure 6:**
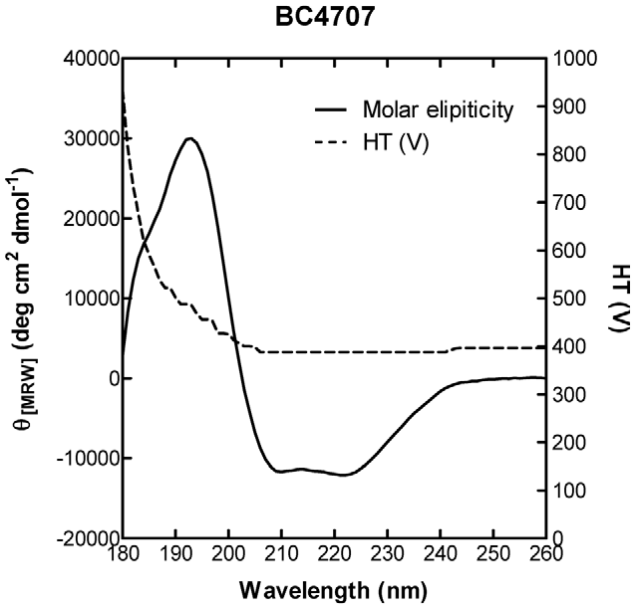
SRCD analysis of purified BC4707-RGSHis_6_ protein. CD spectral analysis of BC4707-RGSHis_6_ was performed over 260-170 nm at 1 nm intervals with an integration time of 1 s. The spectrum shown is buffer subtracted and is an average of 10 scans. The dashed line represents the HT voltage at the photomultiplier.

The integrity of the purified protein was further demonstrated by circular dichroism (CD) measurements. Negative bands at 208 nm and 222 nm and a positive band at 193 nm indicated predominantly α-helical secondary structure (Fig. 6), which is consistent with the predicted topology of BC4707 of 14 α-helices (data not shown). These experiments demonstrated that BC4707 was expressed as a full sized RGSHis_6_ fusion protein in *E. coli* and that the protein localized to the membrane verifying the integrity and localization of the protein upon heterologues expression.

### BC4707 is a multidrug transport protein

*E. coli* DH5α *ΔacrAB* contains a deletion in *acrA* encoding a membrane fusion protein and *acrB* encoding a multidrug efflux system protein causing inactivation of the RND-transporter complex, which results in an *E. coli* mutant that is hypersensitive to many toxic compounds compared to the wild type [30]–[33]. This permits sensitive functional characterization of multidrug transporters following their expression from a plasmid [34], [35]. The presence of pBC4707 in *E. coli* DH5α *ΔacrAB* resulted in improved tolerance to 3 of 12 tested xenobiotics compared to the strain carrying the empty vector, pTTQ18 (Table 4). Increased expression of BC4707-RGSHis_6_ by IPTG induction resulted in even higher tolerance to the fluoroquinolones ciprofloxacin and norfloxacin, whereas no further tolerance to kanamycin was observed (Table 4). It is possible that the increased tolerance to kanamycin in the pBC4707 carrying strain is due to leaky expression of BC4707-RGSHis_6_ prior to the addition of kanamycin, which potentiates drug expulsion. The reason there was no further increase in tolerance after IPTG induction could be that since kanamycin and IPTG was added simultaneously inhibition of protein translation by kanamycin could have prevented further increase of BC4707-RGSHis_6_ in the membrane.

**Table 4:** Susceptibilities of *E. coli* DH5α *ΔacrAB* expressing *bc4707* (pBC4707) compared to its vector control (pTTQ18) under uninduced (LB (0 mM IPTG)) and induced (LB (0.05 mM IPTG)) conditions.

	MIC (µg/ml)			MIC (µg/ml)		
	LB (0 mM IPTG)			LB (0.05 mM IPTG)		
	*E. coli* DH5α *ΔacrAB*			*E. coli* DH5α *ΔacrAB*		
Compound	pTTQ18	pBC4707	§	pTTQ18	pBC4707	§
Chloramphenicol	0.3125	0.3125	1	0.3125	0.3125	1
Ciprofloxacin	0.01	0.04	4	0.01	0.16	16
Bile salts	3200	3200	1	3200	3200	1
Deoxycholate	1600	1600	1	1600	1600	1
SDS	50	50	1	50	50	1
Crystal violet	0.25	0.25	1	0.25	0.25	1
Kanamycin	2.5	10	4	2.5	10	4
Nalidixic acid	30	30	1	30	30	1
Erythromycin	3.13	3.13	1	3.13	3.13	1
Ethidium bromide	2.5	2.5	1	2.5	2.5	1
Norfloxacin	0.08	0.32	4	0.08	1.28	16
Tetracycline	1.56	1.56	1	1.56	1.56	1

§ represents the fold difference in MIC between the *E. coli* DH5α *ΔacrAB* (pBC4707) strain compared to the vector control (DH5α *ΔacrAB* (pTTQ18)).

### Transport by BC4707 utilizes metabolic energy

**Figure 7:**
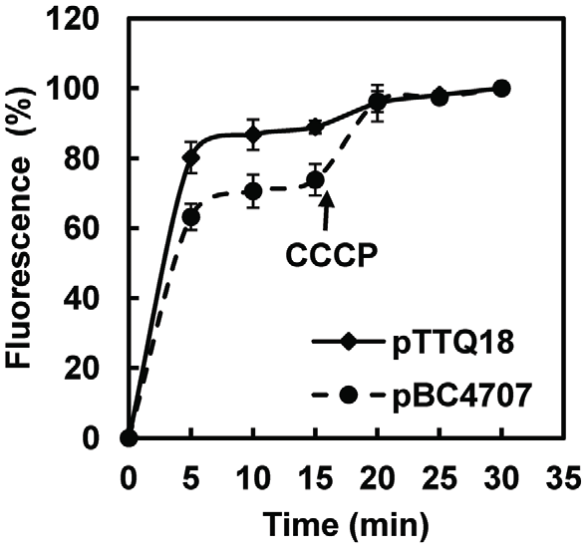
Accumulation of Norfloxacin in *E. coli* DH5α *ΔacrAB* (pBC4707) and DH5α *ΔacrAB* (pTTQ18). The fluorescence of intracellularly accumulated norfloxacin was measured at 280 nm excitation and 445 nm emission in the supernatant of bacterial extracts. Bacteria expressing BC4707-RGSHis_6_ are indicated by circles whereas bacteria carrying the empty vector are shown as diamonds. The assay was started by the addition of 100 µM norfloxacin at time 0 min. At 15 min, the protonophore CCCP (100 µM) was added to disrupt the proton motive force. Shown are averages and standard deviations from two independent experiments.

The classification of BC4707 as an MFS protein implies that it is a secondary active transporter for efflux driven by the cation gradient, probably H^+^ built up during cell respiration [36]. To test if the function of BC4707 is dependent on an intact proton gradient a norfloxacin accumulation assay was performed in *E. coli* (Fig. 7). Cells expressing BC4707-RGSHis_6_ had lower fluorescence intensity compared to cells carrying the empty plasmid pTTQ18, indicating that the presence of BC4707-RGSHis_6_ prevents accumulation of norfloxacin in the cells, most likely via active efflux of the drug (Fig. 7). Carbonyl cyanide 3-chlorophenylhydrazone (CCCP) is a protonophore that destroys the proton gradient [37]–[39]. Addition of CCCP to the assay buffer resulted in increased accumulation of norfloxacin in cells, and the increase in accumulation is relatively more pronounced in the cells expressing BC4707-RGSHis_6_ (Fig. 7), indicating that efflux mediated by BC4707-RGSHis_6_ is dependent on an intact proton gradient. Taken together these data demonstrate that BC4707, as predicted by *in silico* analysis, is a multidrug efflux protein driven directly or indirectly by the proton motive force.

### Transcription analyses demonstrate that the expression level of *bc4707* is not altered in response to sub-lethal concentrations of selected drugs

**Figure 8:**
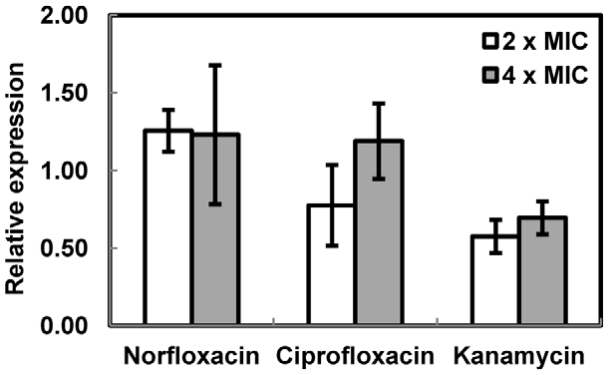
Relative expression of *bc4707* in stressed cells compared to unstressed cells. The expression level of *bc4707* was measured by qRTPCR and was normalized against the expression of16S RNA. The values shown are ratios of the normalized expression levels of cells stressed with antibiotics for 15 min compared to unstressed cells. Shown are averages and standard deviations of at least two independent experiemnts each done with two technical replicates.

The susceptibility assay of *E. coli* DH5α *ΔacrAB* indicate that BC4707 has the capability to transport xenobiotics such as fluoroquinolones and possibly kanamycin, whereas the susceptibility assay in *B. cereus* ATCC14579 showed involvement of BC4707 in norfloxacin tolerance. We therefore tested the transcriptional level of BC4707 in *B. cereus* in the presence and absence of the drugs. *B. cereus* ATCC14579 was grown in LB medium to logarithmic phase (OD_600_ of 0.5), norfloxacin, ciprofloxacin or kanamycin were added and the culture was incubated for 15 minutes followed by RNA extraction. Quantitative RT-PCR-analysis (using 16S RNA as reference) indicated that *bc4707* transcription was not significantly affected by stress with these antibiotics compared to an unstressed control (Fig. 8). Similar results were obtained using GatB RNA [40] as reference (data not shown).

## Discussion

Previous results mapping the stress response induced by bile salt in *B. cereus* [21] indicated a role for BC4707 in bile salts tolerance. By contrast, phenotype analyses comparing the response of *B. cereus* ATCC14579 and its isogenic *Δbc4707* mutant to bile salt-induced stress showed that BC4707 is not vital for *B. cereus* under these conditions (Fig. 1 and Table 2). This suggests that the cells either compensate for the absence of BC4707 under bile salt stress or it is a minor contributor to the bile salt protection under the conditions tested. Microarray analyses comparing the bile salts stress responses of the wild type and *Δbc4707* mutant did not reveal major compensatory alterations in gene expression (Table 1). The general conclusion is therefore that BC4707 does not have a significant role in the defense of *B. cereus* against bile salts stress.

Similarly, it has been shown that *bc4707* expression is induced under acetic acid shock [22] but direct comparison of the *Δbc4707* mutant and the wild type failed to demonstrate a major role for BC4707 under this condition (data not shown). Taken together, these experiments show that although transcriptional data suggested participation of BC4707 in protecting *B. cereus* ATCC14579 against bile salt stress and acetic acid stress, which are two conditions that enteropathogenic bacteria theoretically would encounter, it is not essential under these conditions.

Functional characterization of BC4707 in *E. coli* DH5α *ΔacrAB* confirmed the *in silico* prediction that it is a secondary metabolite multidrug transporter (Fig. 7, Table 4). Yet, the deletion of *bc4707* in *B. cereus* failed to confirm a major role in multidrug transport (Table 2). The *Δbc4707* strain had a somewhat increased susceptibility to only one (norfloxacin) of 12 tested xenobiotics compared to the wild type. It is likely that the reason for this discrepancy is due to the vast number of multidrug transporters in *B. cereus*; these may offer elasticity to the antibiotic stress response and conceal the contribution of the moderately expressed BC4707 under the tested conditions. Moreover, transcriptional data showed that BC4707 expression is not induced by norfloxacin, ciprofloxacin or kanamycin stress, indicating that BC4707 only protects against these antibiotics when it is already present in the membrane. Therefore it is possible that the involvement of BC4707 in multidrug transport is small under specific conditions, but may be physiologically relevant to the bacteria under other conditions. The effect of *bc4707* inactivation on norfloxacin tolerance in addition to the increased tolerance of *E. coli* DH5α *ΔacrAB* expressing BC4707-RGSHis_6_ suggests that BC4707 is involved in protecting the cells against the action of at least some fluoroquinolone compounds. The potential involvement of membrane transport proteins in fluoroquinolone resistance by members of the *Bacillus cereus* group of bacteria is becoming increasingly evident. It was recently reported that during ciprofloxacin stress of *B. anthracis*, the gene GBAA0834, a TetR type transcriptional regulator was a hotspot for mutations leading to ciprofloxacin resistance. As a consequence of mutations leading to disruption of GBAA0834, several adjacent multidrug transporters were up regulated and were probably responsible for the increased ciprofloxacin tolerance [41]. These findings are of concern, since fluoroquinolones are the first line of antimicrobial compounds used in the treatment of *B. anthracis* infections [42], [43].

Although norfloxacin transport is an important function for BC4707 from a clinical perspective, it is likely not the original native function. A BLAST search showed that chromosomally encoded orthologues of BC4707 with high amino acid identity (>91%) exist in all 88 members of the *B. cereus* group of bacteria that have been sequenced to date (data not shown), indicating this gene as being part of the core genome with an ancient and conserved function. Fluoroquinolone compounds, on the other hand are synthetic drugs, first introduced in the early 1960s. Multidrug transporters have been shown to have additional fundamental physiological functions in addition to the multidrug transport capability [17]–[19], [23]–[25]. Furthermore, it has been speculated that environmental stress from conditions in the natural habitat of bacteria may be responsible for the conservation of multidrug transporters and the spread of multidrug resistant bacteria [44]. It is likely that the fluoroquinolone transport capability of BC4707 is an additional effect to the original native function. Based on this it was hypothesized that the reason BC4707 is conserved in all members of the B. cereus group is due to an indigenous function that is important in the natural environment of the bacteria.

Attempts using phenotype experiments comparing the wild type and *Δbc4707* mutant under a range of stressful conditions, including metabolic stress, different salt conditions and pH, failed to elucidate an essential indigenous role of BC4707 in the normal physiology of *B. cereus*. The microarray data from comparison of the wild type and *Δbc4707* mutant during exponential growth in rich medium showed differential regulation of a set of genes involved in metabolism of cysteine, as well as alternative carbon sources (Table 3). We hypothesized that the differential regulation of these genes may reflect a compensatory mechanism to counteract accumulation of toxic metabolites. It is possible that this compensation is enough to sustain normal growth of the mutant in the absence of BC4707. Alternatively, the difficulty in identifying an alternative role for BC4707 in addition to multidrug transport is due to redundancy among the encoded multidrug transporters at the conditions tested. It is likely that inactivation of additional efflux proteins is required in order to clarify the role of BC4707 in the normal physiology of *B. cereus* as well as in the potential multidrug transport.

## Materials and Methods

Bacterial strains and plasmids used in this study are listed in table 5. If not otherwise stated, culture conditions were LB medium at 30°C and 220 rpm.

**Table 5:** Strains and plasmids used in this study.

Strains or plasmids	Relevant genotype or description	Source or reference
***Bacillus cereus*** ** strains**		
ATCC14579	Wild type	Laboratory collection
AH1598	ATCC14579 Δ*bc4707* (pBClin15^−^)	This study
AH1709	ATCC14579 (pBclin15^−^)	This study
***Escherichia coli*** ** strains**		
BL21 (DE3)	F^−^ *ompT hsdS*_B_(r_B_^−^ m_B_^−^) *gal dcm* (DE3)	Novagen
DH5α	*endA1 hsdR17 supE44 thi-1 recA1 gyrA relA1* Δ (*lacZYA-argF*)*U169* (*φ*80*lacZ*ΔM15)	Laboratory collection
AH1805	DH5α *ΔacrAB*	This Study
**Plasmids**		
pTTQ18	E. coli expression plasmid, Amp^r^	[26]
pBC4707	pTTQ18[PlacI^Q^/bc4707]	This study
pBKJ236	E. coli-Bacillus shuttle vector, Ery^r^, Ori(Ts)	[46]
pBKJ236_bc4707	pBKJ236[*Δbc4707*]	This study
pBKJ223	[Pamy/IsceI], Tet^r^	[46]

### Construction of mutants

The *E. coli* DH5α *ΔacrAB* mutant was constructed according to the λ-Red recombinase based method described by Datsenko and Wanner [45]. Briefly, approximately 300 ng of processed PCR product was introduced by electroporation into *E. coli* DH5α containing pKD46, which expresses λ-Red recombinase. The PCR product was amplified from pKD3 and consists of a chloramphenicol cassette flanked by FRT-sites and approximately 40 bp homologous to the 5′ end of *acrA* as well as the 3′ end of *acrB*. Primers used in the study are listed in Table 6. Recovered colonies carrying the chloramphenicol cassette in place of *acrAB* were purified twice on LB agar containing chloramphenicol (20 µg/ml). A markerless mutant was constructed by introducing the pCP20 plasmid encoding a FLP-recombinase into the chloramphenicol resistant mutant followed by screening for antibiotic sensitive mutants. Recovered colonies were purified twice on LB agar. The mutation was confirmed by sequencing.

**Table 6:** Primers used in this study.

Purpose and name	Sequence
**Construction of KO-mutants**	
***B. cereus*** ** ATCC14579**	
AV19_upBC4707_Xba_F_II	CTATCTAGAATTTCGGCCTATCTGGACCT
AV20_upBC4707_SalI_R_II	ATAGTCGACCATTTGTTTTCCCCTTACCC
AV9_downBC4707_SalI_F_new	ATGTCGACTAAAAAGGATGACCATTTTGGTCATCCT
AV10_downBC4707_PstI_R_new	ATACTGCAGCCATTTGGGCCATCACCATGTAA
***E. coli*** ** DH5α**	
acrAB-KO-Start	ATGAACAAAAACAGAGGGTTTACGCCTCTGGCGGTCGTTCGTGTAGGCTGGAGCTGCTTC
acrAB-KO-Stop	TGTGCTCGATATCTTCATTCTTGCGGCTAAAGCGGCGGCGCATATGAATATCCTCCTTAGT
**Sequencing of pBKJ236Δ** ***bc*** **4707**	
pBKJ236_MCS_1	ACACTTTATGCTTCCGGCTC
pBKJ236_MCS_2	CAGTCTATCCCCTGGCGAA
**Sequencing of KO-mutants**	
***Bacillus cereus Δbc4707***	
AV55_forwKO4707_1CO	GTGACCGCTCATTACAAGGTC
AV59_revKO4707_2CO	ATGTTGTAACGCCGAGTAAAGG
***E. coli*** ** Dh5α ** ***ΔacrAB***	
acrAB-KO-control-for	CATCGAGGATGTGTTGGC
acrAB-KO-control-rev	TTTGGGTGAGTATTCATCCAT
**Construction of pBC4707**	
Bc4707-Forward_EcoRI	CCGGAATTCGCATATGAGAAAAAAAGTCATGATGTCTTTAATGTT
Bc4707-Reverse_PstI	AAACTGCAGCTTGTTGCTCACTTGTTTGTGTAGCTGGCTTTAATA
**Sequencing of pBC4707**	
pTTQ18-MCS-start	TTGCGCCGACATCATAACG
pTTQ18-MCS-stop	CCTCTTCGCTATTACGCCA

A markerless knock-out mutant in *B. cereus* ATCC14579 was constructed essentially as described by Janes & Stibitz [46]. Briefly, the fused up- and downstream regions flanking *bc4707* were amplified by PCR and introduced into the suicide shuttle vector pBKJ236. The vector contains the recognition site for the homing endonuclease I-*Sce*I. After transformation, pBKJ236 was incorporated into the chromosome of *B. cereus* via homologous recombination. Selection of recombinants were done on LB plates containing erythromycin (5 µg/ml) and was facilitated by the temperature sensitive origin of replication in pBKJ236 which does not permit replication of the plasmid at 37°C. Following transformation of a second plasmid (pBKJ223) carrying the gene encoding the homing endonuclease I-SceI, double strand breaks were introduced in the chromosome at the site of the inserted plasmid. The double strand breaks force homologous recombination events to repair the DNA damage which results in either wild type or mutant genotypes. Recombinants that had undergone the second recombination step were identified as erythromycin sensitive colonies. Screening for markerless mutants was done by PCR amplification using primers annealing up- and downstream of *bc4707*. The mutation was confirmed by sequencing.

### Construction of the *E. coli* expression vector pBC4707

The gene *bc4707* was amplified by PCR from genomic DNA extracted from the *B. cereus* type strain ATCC14579 using the primers Bc4707-forward_EcoRI and Bc4707-reverse_PstI (Table 6). The PCR product was cloned into the plasmid pTTQ18 by restriction digestion using EcoRI and PstI and ligation using T4-DNA ligase (New England Biolabs). Ampicillin resistant colonies were clean streaked twice and the vector insert was verified by sequencing.

### Expression of BC4707-RGSHis_6_ and preparation of membrane fractions

Optimization of protein expression in *E. coli* BL21 (DE3) (pBC4707) was done in 100 ml cultures at 37°C, 150 rpm. IPTG (0–1 mM) was added to the cultures in the mid-logarithmic growth phase (OD_600_ of 0.4–0.6). Protein expression was induced for 3 h. The bacterial cells were disrupted by the water-lysis method [47] and the membrane fraction was prepared as described previously [47]. For large scale production of BC4707-RGSHis_6_, *E. coli* BL21 (DE3) (pBC4707) was grown in a 30 liter culture in a fermenter at 37°C. Protein expression was induced for 3 h following addition of 1 mM IPTG at OD_600_ 0.4–0.6. Bacterial cell disruption was achieved by explosive decompression in a Constant Cell Disruption System (WWW.constantsystems.com). Inner membranes were prepared as previously described [47].

### Solubilization and purification of the BC4707-RGSHis_6_ membrane protein

*E. coli* inner membranes containing overexpressed BC4707-RGSHis_6_ were added to solubilization buffer [20 mM Tris pH 8.0, 30 mM imidazole pH 8.0, 300 mM NaCl, 20% glycerol, 1% dodecyl-beta-D-maltoside (DDM)] to give a final protein concentration of 3 mg/ml. The suspension was incubated at 4°C for a minimum of 1 hour with gentle mixing. Insoluble proteins were removed by ultracentrifugation at 100,000× g for 1 hour at 4°C. The solubilized protein was added to the Ni-NTA resin (Qiagen™, Germany) and incubated for a minimum of 1.5 hours at 4°C with gentle mixing. The unbound protein was allowed to run off and the protein-bound resin was washed with 10× resin volumes of wash buffer (20 mM Tris pH 8.0, 30 mM imidazole, 10% glycerol, 150 mM NaCl, 0.05% DDM) and eluted using imidazole elution buffer (20 mM Tris pH 8.0, 200 mM imidazole, 5% glycerol, 150 mM NaCl, 0.05% DDM). The eluted protein was concentrated using a Vivaspin 20 (100 kDa MWCO) concentrator (Sartorius Ltd., Epsom, UK) and desalted using a Econo-Pac® 10DG desalting gel column (BioRad, California, USA) to remove any extraneous imidazole remaining from the elution step. The concentrated protein was analyzed immediately or stored at −80°C.

### Preparation and analysis of samples by Synchrotron Radiation Circular Dichroism spectroscopy

Purified BC4707-RGSHis_6_ protein was resuspended to a final concentration of 0.5 mg/ml in CD buffer (10 mM potassium phosphate, pH 7.6; 0.05% DDM) and the sample placed into a 0.02 cm pathlength circular cell. CD measurements were performed on Beamline B23 (Diamond Light Source, Didcot, UK) at 18°C. Data points were recorded at over 260-170 nm at 1 nm intervals with an integration time of 1 s. A spectrum of the CD buffer was taken and subtracted as background.

### RNA isolation

Bacterial cells were harvested by diluting the cultures with an equal volume of ice-cold methanol followed by centrifugation at 4,000 *g* for 5 min at 4°C. Cells were disrupted using a Precellys®24 tissue homogenizer. RNA isolation was performed using the RNeasy Mini Kit (Qiagen, Germany) according to the manufacturer's protocol, including the optional on column DNAse treatment. After elution, the RNA was treated with Turbo DNAse (Applied Biosystems, USA) according to the manufacturer's manual followed by a second round of purification using the RNeasy Mini Kit. The concentration of RNA was determined by UV-spectrometry and the quality was controlled by agarose gel electrophoresis as well as UV-spectrometry.

### Quantitative RT-PCR

Complementary strand DNA was synthesized from 1 µg of RNA using the Superscript III reverse transcriptase (Invitrogen) according to the manufacturer's instructions. The cDNA was diluted 1∶5 (1∶2500 for the reference gene 16S). Three microliter of diluted cDNA was mixed with primers (0.5 µM) as well as qPCR&GO™ LC480 green master mix (MPBiomedicals, USA) to a final volume of 15 µl. Primers were designed to result in an amplicon of approximately 100 bp (Table 6). Quantitative PCR was performed with a Roche Lightcycler 480 (Roche Diagnostics GmbH, Germany). The cycling conditions were 95°C for 1.5 min followed by 45 cycles at 95°C for 10 seconds, 58°C for 10 seconds and 72°C for 10 seconds. The crossing point (Ct) values were determined by the 2^nd^ derivative maximum of two technical replicates per biological replicate. Results were calculated by the ΔΔCt approximation. Expression ratios are averages of at least 2 biological replicates including 2 technical replicates using 16S as the reference gene for normalization. In addition, GatB RNA was used as reference to confirm the results.

### Microarray analysis

The microarray slides, containing 70-mer oligonucleotides representing all the genomic ORFs of *B. cereus* (ATCC14579), were printed at the Microarray core facility of the Norwegian University of Science and Technology (NTNU) in Trondheim. The microarray experiments were conducted as previously described by Gohar *et al.* [48]. Briefly, the microarray slides were pre-incubated at 42°C for 45 minutes in pre-hybridization buffer (5×SSC, 0.1% SDS, 0.1% BSA); washed in MQ water followed by isopropanol and finally spun dry. Complementary strand DNA synthesis, labeling and purification was carried out with the FairPlay III™ microarray labeling kit (Stratagene, USA), using 500 ng random hexamers (Applied Biosystems, USA), 20 µg of RNA, and Cy3 or Cy5 dyes (GE Healthcare Bio-Sciences AB, Sweden). After purification, the samples were concentrated with a Microcon column (Millipore, USA). Labeled DNA was mixed with hybridization solution (30% formamide, 5×SSC, 0.1% SDS and 0.1 mg/ml salmon sperm DNA) denatured at 95°C for 2 minutes, and incubated at 42°C before hybridization. Hybridization was carried out over night at 42°C in a hybridization chamber (Monterey Industries, CA, USA) humidified with 5×SSC. After hybridization, the slides were washed at 42°C in a buffer containing 0.5×SSC and 0.01% SDS followed by 0.06×SSC, and finally in isopropanol before they were spun dry. The slides were scanned with an Axon 4000B scanner using the GenePix Pro 6.0 software (Molecular Devices, CA, USA). Microarray data was analyzed with a custom R-script in R 2.10.1 [49]. The GenePix files were imported to R, and the Limma package [50] was used for filtering, normalization, and further analysis. Microarray experiments were conducted with two independent experiments analyzed in two dye swapped technical replicates each. The Microarray data are available in the ArrayExpress database (www.ebi.ac.uk/arrayexpress) under accession numbers E-MEXP-3529, E-MEXP-3535, E-MEXP-3536 and E-MEXP-3537.

### Susceptibility assays

Bacteria were grown in LB medium at 30°C, 220 rpm overnight, diluted 1∶100 into a pre-culture and incubated at the same conditions until mid-logarithmic phase (OD_600_∼0.5). The pre-culture was diluted to OD_600_ of 0.02 and distributed in 100 µl aliquots in the wells of 96 wells plates (Becton Dickinson Labware, Franklin Lakes, USA). Compounds were added to the wells at appropriate concentrations in 2× dilution series from 100× concentrated stock solutions. For susceptibility assays using *E. coli* DH5α *ΔacrAB* carrying pTTQ18 or pBC4707, ampicillin (100 µg/ml) was included in the medium. These assays were carried out in protein expression uninduced (0 mM IPTG) as well as induced (0.05 mM IPTG) conditions. The plates were incubated at 30°C, 220 rpm for 20 h and then analyzed by ocular inspection as well as by a Victor™×multilabel plate reader (Perkin-Elmer) at 595 nm. Minimum inhibitory concentrations were determined as the concentration of xenobiotics at which the OD after 20 h incubation was less than double the starting OD. In case visual inspection showed aggregated cells at the bottom of the wells in otherwise transparent media, the OD measurements were considered unreliable whereas the MIC value was determined as the concentration where the media turned from turbid to transparent. In most cases, the two methods to determine the MIC correlated.

### Growth in GGGS medium

Bacterial growth under nutrient poor conditions was tested using a minimal medium derived from GGGS medium [51]. The composition of the medium was K_2_HPO_4_ (3.0 mM), KH_2_PO_4_ (3.5 mM), MgSO_4_ (0.8 mM), MnCl_2_ (0.04 mM), NaCl (0.2 mM), CaCl_2_ (0.2 mM), ZnCl_2_ (0.05 mM), and FeCl3 (0.04 mM). In addition to this, 2 mM nitrogen source as well as 20 mM carbon source was added. Combinations of 7 different nitrogen sources and 6 different carbon sources were tested. The carbon sources investigated were: glucose, lactate, maltose, sorbitol, galactose, pyruvate, and xylose. The nitrogen sources investigated were: L-serine, L-glutamate, L-arginine, L-proline, urea and ammonium chloride. Bacteria were grown overnight in LB medium at 30°C and 220 rpm shaking. The culture was diluted 1∶100 in fresh LB medium and cultured under the same conditions until mid-logarithmic phase. The bacteria were washed once in GGGS medium and resuspended in fresh GGGS medium including nitrogen and carbon sources to an OD_600_ of 0.01. Initially, attempts were made to establish growth curves for the mutant and wild type. The cultures were incubated in 100 ml Erlenmeyer flasks filled to a fourth of the volume, at 220 rpm at 30°C for up to 140 h. The absorbance was measured at regular intervals. In a subsequent experiment the bacterial suspensions (OD_600_ 0.01 in GGGS medium) were spotted onto 1.5% agar plates containing GGGS medium supplemented with nitrogen and carbon sources. These plates were incubated at 30°C for 140 h. Bacterial growth was scored depending on the density and appearance of the colonies.

### Motility assays

Swimming motility was observed on 0.3% LB agar plates whereas swarming motility was observed on 1.0% LB agar plates supplemented with 0.5% glucose. The plates were inoculated with 5 µl of culture (OD_600_∼5) that had been concentrated from a logarithmically growing culture (OD_600_∼1). Plates were incubated at 37°C. The diameter of the zone created by the swimming bacteria and the radius of the zone created by the swarming bacteria respectively were measured at regular intervals for 70 h. Similar experiments were conducted using 0.3% and 1.0% GGGS agar plates supplemented with different combinations of carbon and nitrogen sources as described above.

### Phenotype microarray

Bacteria were inoculated onto tryptic soy agar (TSA), incubated at 28°C over night and clean streaked onto TSA for an additional over night incubation. Single colonies, picked using sterile cotton swabs, were suspended in inoculation fluid (IF-0, Biolog, Hayward, CA, USA). The transmittances of the bacterial suspensions were adjusted to 85% on a Biolog turbidometer. Cell suspension, inoculation fluids, tetrazolium dye, and suitable additives were mixed according to the manufacturer's protocol optimized for *Bacillus subtilis*. The mixture was inoculated into the Phenotype Microarray plates (100 µl per well), which were incubated at 28°C in an Omnilog reader. Quantitative color changes were recorded every 15 min by a charge-coupled camera device for a period of 48 h. The kinetic responses were analyzed with the Omnilog-PM software. The data was analyzed visually as well as by comparing the calculated areas under the kinetic response curves.

### Norfloxacin accumulation assay

Bacterial cells for the norfloxacin accumulation assay were prepared by growing *E. coli* DH5α *ΔacrAB* (pBC4707) as well as *E. coli* DH5α *ΔacrAB* (pTTQ18) at 37°C in LB broth containing ampicillin (100 µg/ml) over night. The cultures were diluted to an OD_600_ of 0.05 in fresh LB broth including ampicillin (100 µg/ml). These cultures were grown to OD_600_∼0.5, at which point IPTG was added to a concentration of 0.1 mM. Expression of proteins was induced for 3 hours. Following this, the cells were spun down, washed twice in Tris-Cl (pH 7.0) including 100 mM NaCl and resuspended in the same buffer. The OD_600_ was adjusted to 1 and glucose was added to a final concentration of 20 mM. The assay was started by the addition of 100 µM norfloxacin. Samples (1 ml) were taken every five minutes for 30 min starting before (time 0 min) the addition of norfloxacin. CCCP (100 µM) was added at time 15 min. Samples were centrifuged at 13000 rpm for 30 seconds, the supernatant was discarded. The bacterial pellet was washed once in Tris-Cl (pH 7.0) including 100 mM NaCl and resuspended in 0.1 M glycin (pH 3.0). The Norfloxacin was extracted over night at 37°C. The bacterial debris was removed by centrifugation and the fluorescence of the supernatant was measured at 280 nm excitation and 445 nm emission on a Perkin-Elmer LS-55 fluorescence spectrophotometer.

### Acid stress

Acid stress experiments were conducted basically as described by Mols et al. [22]. Pre-cultures of the *B. cereus* wild type as well as the *Δ4707* isogenic mutant were inoculated from overnight cultures. The pre-cultures were grown until mid-logarithmic phase (OD_600_ was between 0.5–1.0) at which point they were diluted 1∶100 in brain heart infusion medium (BHI) buffered to pH 7.1 with 100 mM or 10 mM sodium phosphate. The buffered cultures were grown to an OD_600_ of 0.5. At this point the medium was acidified to pH 5.5 using 0.238% (v/v) 12 M hydrochloric acid, 0.69% (v/v) lactic acid, 0.205% (v/v) 12 M hydrochloric acid with 0.074% (v/v) acetic acid or 0.571% (v/v) acetic acid, respectively. The OD_600_ of the cultures were recorded for an additional 4.5 h.

## Supporting Information

Table S1 **Bile salt induced transcriptional profiles of** ***B. cereus*** **ATCC14579 and its isogenic** ***Δbc4707*** **mutant.** Bacteria were grown to OD_600_ 0.5 (t^OD 0.5^) at which point bile salts (50 µg/ml) were added followed by additionally 15 min incubation (t^BS 15^). Cells were harvested and total RNA was prepared from each strain before (t^OD 0.5^) and after (t^BS 15^) bile salts stress. Microarray analysis was conducted comparing the gene expression at time t^BS 15^ with time t^OD 0.5^. Shown are genes with significantly (P<0.05) different relative expression (t^BS 15^/t^OD 0.5^>1.5 or t^BS 15^/t^OD 0.5^<0.67) in at least one strain after bile salts stress for 15 min. * indicates relative expression values that were not statistically significant (i.e. p>0.05).
(DOC)
