# Supplementary material for: BC4707 Is a Major Facilitator Superfamily Multidrug Resistance Transport Protein from Bacillus cereus Implicated in Fluoroquinolone Tolerance
Source: PLoS One. 2012 May 16;7(5):e36720. doi: 10.1371/journal.pone.0036720 (PMC3353944; doi:10.1371/journal.pone.0036720)
Supplement: Table S1 — Bile salt induced transcriptional profiles of B. cereus ATCC14579 and its isogenic Δbc4707 mutant. Bacteria were grown to OD600 0.5 (tOD 0.5) at which point bile salts (50 µg/ml) were added followed by additionally 15 min incubation (tBS 15). Cells were harvested and total RNA was prepared from each strain before (tOD 0.5) and after (tBS 15) bile salts stress. Microarray analysis was conducted comparing the gene expression at time tBS 15 with time tOD 0.5. Shown are genes with significantly (P<0.05) different relative expression (tBS 15/tOD 0.5>1.5 or tBS 15/tOD 0.5<0.67) in at least one strain after bile salts stress for 15 min. * indicates relative expression values that were not statistically significant (i.e. p>0.05). (DOC) [file pone.0036720.s001.doc]

**Table S1 Bile salt induced transcriptional profiles of *B. cereus* ATCC14579 and its isogenic *Δbc4707* mutant.** Bacteria were grown to OD_600_ 0.5 (t^OD 0.5^) at which point bile salts (50 µg/ml) were added followed by additionally 15 min incubation (t^BS 15^). Cells were harvested and total RNA was prepared from each strain before (t^OD 0.5^) and after (t^BS 15^) bile salts stress. Microarray analysis was conducted comparing the gene expression at time t^BS 15^ with time t^OD 0.5^. Shown are genes with significantly (P < 0.05) different relative expression (t^BS 15^/t^OD 0.5^ >1.5 or t^BS 15^/t^OD 0.5^< 0.67) in at least one strain after bile salts stress for 15 min. * indicates relative expression values that were not statistically significant (i.e. p>0.05).

|  |  | **Relative expression** | |
| --- | --- | --- | --- |
|  |  | **(t^BS 15^ / t^OD 0.5^)** | |
| **Gene** | **Name** | **Wild type** | **Mutant** |
| *bc0013* | inositol-5-monophosphate dehydrogenase | 1.65 * | 2.05 |
| *bc0015* | pyridoxine biosynthesis protein | 1.73 * | 2.52 |
| *bc0016* | glutamine amidotransferase, SNO family | 1.21 * | 1.77 |
| *bc0021* | deoxynucleoside kinase family protein | 0.37 * | 0.44 |
| *bc0042* | transition state transcriptional regulatory protein AbrB | 1.11 * | 1.59 |
| *bc0043* | methionine--tRNA ligase | 1.38 * | 1.92 |
| *bc0044* | deoxyribonuclease, TatD family | 1.15 * | 1.84 |
| *bc0053* | stage V sporulation protein G | 1.28 * | 1.62 |
| *bc0070* | hypothetical protein | 1.31 * | 2.01 |
| *bc0071* | hypoxanthine-guanine phosphoribosyltransferase | 1.74 * | 2.32 |
| *bc0072* | cell division protein FtsH | 1.79 * | 2.76 |
| *bc0073* | transcriptional activator, putative, Baf family | 1.31 * | 1.99 |
| *bc0074* | Hsp33-like chaperonin | 1.36 * | 1.72 |
| *bc0075* | cysteine synthase A | 1.17 * | 1.81 |
| *bc0076* | para-aminobenzoate synthase component I | 1.26 * | 1.76 |
| *bc0077* | para-aminobenzoate synthase component II | 1.50 * | 2.01 |
| *bc0078* | 4-amino-4-deoxychorismate lyase | 1.38 * | 1.72 |
| *bc0079* | dihydropteroate synthase | 1.31 * | 1.77 |
| *bc0081* | 2-amino-4-hydroxy-6- hydroxymethyldihydropteridine pyrophosphokinase | 1.23 * | 1.63 |
| *bc0099* | transcriptional regulator CtsR | 19.34 | 11.24 |
| *bc0100* | hypothetical protein | 19.02 | 8.10 |
| *bc0101* | putative ATP:guanido phosphotransferase | 10.71 | 7.39 |
| *bc0102* | negative regulator of genetic competence ClpC/MecB | 20.43 | 15.07 |
| *bc0104* | DNA-binding protein, putative | 2.15 | 1.46 * |
| *bc0118* | 50S ribosomal protein L1 | 0.49 * | 0.66 |
| *bc0119* | 50S ribosomal protein L10 | 0.53 * | 0.60 |
| *bc0120* | 50S ribosomal protein L7/L12 | 0.46 * | 0.42 |
| *bc0124* | hypothetical protein | 0.34 | 0.36 |
| *bc0125* | 30S ribosomal protein S12 | 0.88 * | 0.66 |
| *bc0126* | 30S ribosomal protein S7 | 0.85 * | 0.65 |
| *bc0130* | 30S ribosomal protein S10 | 0.68 * | 0.49 |
| *bc0133* | 50S ribosomal protein L23 | 0.65 * | 0.63 |
| *bc0134* | 50S ribosomal protein L2 | 0.64 * | 0.60 |
| *bc0135* | SSU ribosomal protein S19P | 0.81 * | 0.51 |
| *bc0136* | 50S ribosomal protein L22 | 0.78 * | 0.60 |
| *bc0137* | 30S ribosomal protein S3 | 0.60 * | 0.52 |
| *bc0138* | 50S ribosomal protein L16 | 0.72 * | 0.63 |
| *bc0139* | 50S ribosomal protein L29 | 0.72 * | 0.54 |
| *bc0141* | 50S ribosomal protein L14 | 0.80 * | 0.60 |
| *bc0142* | 50S ribosomal protein L24 | 0.73 * | 0.64 |
| *bc0143* | 50S ribosomal protein L5 | 0.70 * | 0.55 |
| *bc0146* | 50S ribosomal protein L6 | 0.86 * | 0.60 |
| *bc0149* | 50S ribosomal protein L30 | 0.74 * | 0.55 |
| *bc0162* | cobalt transport protein | 0.40 | 0.40 |
| *bc0163* | tRNA pseudouridine synthase A | 0.43 | 0.48 |
| *bc0164* | 50S ribosomal protein L13 | 0.65 * | 0.53 |
| *bc0165* | 30S ribosomal protein S9 | 0.48 | 0.50 |
| *bc0168* | mrp protein | 1.62 * | 1.87 |
| *bc0185* | arginase | 2.50 * | 2.01 |
| *bc0217* | oxidoreductase, aldo/keto reductase family | 1.90 * | 2.60 |
| *bc0218* | pyrroline-5-carboxylate reductase | 4.08 | 2.89 |
| *bc0219* | transporter, putative | 0.41 * | 0.57 |
| *bc0246* | hydrolase (HAD superfamily) | 1.39 * | 1.59 |
| *bc0248* | hypothetical protein | 2.47 | 3.04 |
| *bc0250* | hypothetical protein | 3.55 | 2.24 * |
| *bc0251* | hypothetical protein | 3.39 | 2.36 |
| *bc0259* | ATP-dependent RNA helicase, DEAD/DEAH box family | 0.35 | 0.50 |
| *bc0291* | redox-sensing transcriptional repressor Rex | 2.00 | 1.19 * |
| *bc0294* | co-chaperonin GroES | 15.84 | 19.25 |
| *bc0295* | chaperonin GroEL | 11.55 | 13.36 |
| *bc0304* | FrnE protein | 2.37 | 1.67 |
| *bc0344* | 1-pyrroline-5-carboxylate dehydrogenase | 6.76 | 10.61 |
| *bc0360* | aminopeptidase AmpS | 3.17 | 3.04 |
| *bc0369* | hypothetical protein | 1.21 * | 0.62 |
| *bc0376* | alkyl hydroperoxide reductase, F subunit | 1.73 * | 2.43 |
| *bc0377* | alkyl hydroperoxide reductase, subunit C | 2.12 | 3.03 |
| *bc0378* | 5-methylthioribose kinase, putative | 1.62 * | 1.72 |
| *bc0400* | fatty acid desaturase | 0.24 | 0.24 |
| *bc0439* | prolyl-tRNA synthetase | 2.32 | 1.30 * |
| *bc0442* | tellurium resistance protein, putative | 5.33 | 5.96 |
| *bc0443* | tellurium resistance protein | 4.96 | 5.47 |
| *bc0444* | tellurium resistance protein | 5.33 | 3.79 |
| *bc0445* | tellurium resistance protein, putative | 3.09 | 2.63 |
| *bc0446* | hypothetical protein | 2.71 | 2.11 |
| *bc0447* | tellurite resistance protein, putative | 1.92 | 1.18 * |
| *bc0450* | low molecular weight phosphotyrosine protein phosphatase family protein | 1.99 | 1.69 |
| *bc0452* | ribonuclease BN, putative | 1.49 * | 1.88 |
| *bc0491* | formate acetyltransferase | 0.20 | 0.32 |
| *bc0492* | pyruvate formate-lyase-activating enzyme | 0.22 | 0.37 * |
| *bc0494* | hypothetical Cytosolic Protein | 0.43 | 0.56 |
| *bc0508* | hypothetical protein | 2.02 | 1.49 |
| *bc0513* | ABC transporter, ATP-binding protein | 2.33 * | 1.75 |
| *bc0515* | ABC transporter, permease protein, putative | 2.49 | 1.44 * |
| *bc0542* | hypothetical protein | 2.09 | 1.86 |
| *bc0558* | SPFH domain/band 7 family protein | 1.84 * | 1.62 |
| *bc0587* | acetyltransferase, GNAT family | 2.30 | 2.61 |
| *bc0590* | hypothetical protein | 1.35 * | 1.92 |
| *bc0593* | amino acid permease family protein | 1.84 * | 3.00 |
| *bc0597* | nicotinate phosphoribosyltransferase | 1.16 * | 2.39 |
| *bc0598* | Transcriptional activator NprR | 1.53 * | 2.13 |
| *bc0611* | aspartate ammonia-lyase | 1.28 * | 2.14 |
| *bc0612* | L-lactate permease | 0.18 | 0.40 |
| *bc0621* | 8-amino-7-oxononanoate synthase | 3.00 | 2.77 |
| *bc0622* | hypothetical protein | 1.89 * | 2.93 |
| *bc0631* | PTS system, trehalose-specific IIBC component | 0.35 | 0.62 * |
| *bc0632* | trehalose-6-phosphate hydrolase | 0.43 | 0.67 * |
| *bc0641* | amino acid ABC transporter, permease protein | 1.90 | 2.57 |
| *bc0656* | glycerol-3-phosphate transporter | 1.27 * | 1.81 |
| *bc0657* | transcriptional regulator, MarR family | 6.65 | 7.03 |
| *bc0658* | Multidrug resistance protein B | 14.65 | 12.22 |
| *bc0666* | immune inhibitor A metalloprotease | 3.14 * | 5.47 |
| *bc0668* | alcohol dehydrogenase, zinc-containing | 3.98 | 5.47 |
| *bc0670* | phospholipase C | 0.89 * | 1.64 |
| *bc0671* | sphingomyelinase C | 0.92 * | 1.69 |
| *bc0684* | proton/peptide symporter family protein | 3.83 | 2.33 |
| *bc0739* | hypothetical protein | 1.88 * | 2.33 |
| *bc0753* | potassium-transporting ATPase subunit A | 1.71 * | 2.11 |
| *bc0754* | potassium-transporting ATPase subunit B | 1.72 * | 2.86 |
| *bc0759* | phospholipase, putative | 1.70 * | 2.31 |
| *bc0800* | hypothetical protein | 2.20 * | 2.69 |
| *bc0805* | hypothetical protein | 1.82 * | 2.57 |
| *bc0813* | hypothetical protein | 0.68 * | 0.55 |
| *bc0815* | ABC transporter, ATP-binding protein | 0.26 | 0.71 * |
| *bc0883* | alpha-acetolactate synthase | 1.82 * | 3.77 |
| *bc0888* | N-acetylmuramoyl-L-alanine amidase, family 2 | 0.44 * | 0.59 |
| *bc0898* | enoyl-CoA hydratase | 2.31 | 2.14 |
| *bc0917* | hypothetical Cytosolic Protein | 1.52 * | 1.81 |
| *bc0925* | hypothetical Membrane Spanning Protein | 3.86 | 4.22 |
| *bc0926* | hypothetical protein | 1.66 * | 1.77 |
| *bc0945* | DNA integration/recombination/invertion protein | 1.20 * | 1.53 |
| *bc0962* | drug resistance transporter, EmrB/QacA family | 7.64 | 2.74 |
| *bc1003* | serine-protein kinase RsbW | 2.92 | 1.72 |
| *bc1004* | sigma factor B | 2.77 | 2.35 |
| *bc1012* | hypothetical protein | 1.36 * | 1.52 |
| *bc1034* | glycerol uptake facilitator protein | 4.44 | 6.05 |
| *bc1035* | glycerol kinase | 2.11 * | 3.72 |
| *bc1036* | glycerol-3-phosphate dehydrogenase, aerobic | 2.05 * | 2.40 |
| *bc1047* | protease production regulatory protein Hpr | 2.13 | 2.01 |
| *bc1049* | HIT family protein | 1.55 * | 1.61 |
| *bc1066* | hypothetical protein | 2.29 | 1.96 |
| *bc1149* | ornithine--oxo-acid transaminase | 3.09 | 3.69 |
| *bc1160* | hypothetical protein | 0.52 * | 0.65 |
| *bc1161* | peptidylprolyl isomerase | 2.09 | 1.98 |
| *bc1165* | hydrolase, haloacid dehalogenase-like family | 1.84 * | 1.78 |
| *bc1168* | ATP-dependent Clp protease, ATP-binding subunit ClpB | 8.43 | 9.72 |
| *bc1173* | 3-oxoacyl-(acyl carrier protein) synthase | 0.26 | 0.32 |
| *bc1174* | 3-oxoacyl-(acyl carrier protein) synthase | 0.24 | 0.24 |
| *bc1179* | Oligopeptide-binding protein oppA | 1.77 * | 1.71 |
| *bc1180* | oligopeptide ABC transporter, permease protein | 4.89 | 2.67 |
| *bc1181* | oligopeptide ABC transporter, permease protein | 6.90 | 5.82 |
| *bc1182* | oligopeptide ABC transporter, ATP-binding protein | 10.17 | 6.50 |
| *bc1183* | oligopeptide ABC transporter, ATP-binding protein | 6.00 | 4.61 |
| *bc1184* | MATE efflux family protein | 5.05 | 3.18 |
| *bc1185* | Oligopeptide-binding protein oppA | 2.39 * | 3.29 |
| *bc1188* | transcriptional regulator Spx | 1.98 | 1.44 * |
| *bc1190* | adaptor protein | 1.33 * | 1.76 |
| *bc1194* | hypothetical protein | 1.98 | 1.77 |
| *bc1195* | protozoan/cyanobacterial globin family protein | 2.09 | 1.91 |
| *bc1216* | enoyl-(acyl carrier protein) reductase | 0.47 * | 0.42 |
| *bc1224* | Acetyltransferase | 4.79 | 5.40 |
| *bc1225* | hypothetical protein | 3.61 | 5.35 |
| *bc1226* | hypothetical protein | 2.76 | 2.85 |
| *bc1231* | Sodium/proline symporter | 1.90 * | 2.00 |
| *bc1232* | anthranilate synthase component I | 4.65 | 2.69 |
| *bc1233* | anthranilate synthase component II | 15.44 | 6.43 |
| *bc1234* | anthranilate phosphoribosyltransferase | 14.57 | 5.20 |
| *bc1235* | indole-3-glycerol-phosphate synthase | 6.34 | 3.63 |
| *bc1236* | N-(5'-phosphoribosyl)anthranilate isomerase | 9.74 | 6.59 |
| *bc1237* | tryptophan synthase subunit beta | 16.55 | 9.72 |
| *bc1238* | tryptophan synthase subunit alpha | 5.89 | 7.37 |
| *bc1246* | pyridine nucleotide-disulfide oxidoreductase, class I | 2.17 | 1.85 |
| *bc1251* | dihydrolipoamide acetyltransferase | 3.04 | 3.51 |
| *bc1252* | 2-oxoglutarate dehydrogenase, E1 component | 4.13 | 3.44 |
| *bc1282* | transcriptional regulator SinR | 1.05 * | 1.85 |
| *bc1302* | transcriptional regulator, GntR family | 1.16 * | 1.73 |
| *bc1312* | maoC family protein | 0.73 * | 1.94 |
| *bc1313* | phaP protein | 1.21 * | 1.57 |
| *bc1314* | PhaQ protein | 1.81 * | 1.66 |
| *bc1316* | phaR protein | 8.31 | 3.88 |
| *bc1317* | 3-ketoacyl-(acyl-carrier-protein) reductase | 9.62 | 4.62 |
| *bc1318* | poly(R)-hydroxyalkanoic acid synthase, class III, PhaC subunit | 1.60 * | 1.61 |
| *bc1320* | sodium transporter family protein | 2.04 | 1.57 |
| *bc1338* | oligoendopeptidase F, putative | 1.35 * | 2.14 |
| *bc1339* | hypothetical protein | 1.12 * | 1.60 |
| *bc1342* | 6-pyruvoyl tetrahydrobiopterin synthase, putative | 0.48 | 0.92 * |
| *bc1435* | hypothetical protein | 17.45 | 9.06 |
| *bc1346* | hypothetical protein | 1.19 * | 2.35 |
| *bc1366* | hypothetical protein | 0.79 * | 1.64 |
| *bc1385* | hypothetical protein | 1.75 * | 1.73 |
| *bc1435* | hypothetical protein | 17.45 | 9.06 |
| *bc1436* | PspA/IM30 family protein | 6.31 | 3.83 |
| *bc1437* | hypothetical protein | 1.01 * | 1.54 |
| *bc1438* | sensor histidine kinase | 0.75 * | 1.53 |
| *bc1443* | methlytransferase, UbiE/COQ5 family | 1.21 * | 2.87 |
| *bc1451* | hypothetical Membrane Associated Protein | NA | 2.37 |
| *bc1461* | site-specific recombinase, phage integrase family | 3.60 | 3.81 |
| *bc1482* | hypothetical protein | 1.47 * | 1.77 |
| *bc1491* | glutamate dehydrogenase | 1.68 * | 2.39 |
| *bc1498* | 30S ribosomal protein S1 | 2.03 | 1.82 * |
| *bc1505* | NAD(P)H-dependent glycerol-3-phosphate dehydrogenase | 0.47 | 0.83 * |
| *bc1515* | nucleoside diphosphate kinase | 2.35 | 2.56 |
| *bc1523* | cytochrome b6 | 1.05 * | 1.61 |
| *bc1528* | hypothetical protein | 1.94 * | 1.65 |
| *bc1533* | methylglyoxal synthase | 1.55 * | 1.73 |
| *bc1540* | 3-methyl-2-oxobutanoate hydroxymethyltransferase | 4.06 | 3.61 |
| *bc1541* | pantoate--beta-alanine ligase | 3.16 | 1.57 |
| *bc1542* | aspartate 1-decarboxylase precursor | 2.53 | 1.65 |
| *bc1548* | endonuclease III | 1.32 * | 0.65 |
| *bc1581* | Mg(2+) P-type ATPase-like protein | 1.47 * | 1.77 |
| *bc1612* | germination protein gerN | 4.42 | 3.77 |
| *bc1657* | flagellin | 1.75 * | 2.17 |
| *bc1658* | Flagellin | 1.79 * | 2.44 |
| *bc1659* | Flagellin | 1.98 * | 2.45 |
| *bc1692* | hypothetical protein | 2.10 | 1.85 |
| *bc1712* | fumarate hydratase | 2.20 | 2.13 |
| *bc1746* | asparagine synthetase AsnA | 1.12 * | 1.55 |
| *bc1760* | 3-oxoacyl-(acyl carrier protein) synthase | 0.90 * | 0.52 |
| *bc1774* | peptide methionine sulfoxide reductase | 1.88 * | 1.75 |
| *bc1788* | Lysophospholipase L2 | 1.25 * | 1.53 |
| *bc1793* | chlorohydrolase | 1.77 | 1.52 |
| *bc1804* | hypothetical protein | 1.01 * | 1.62 |
| *bc1809* | enterotoxin | 1.37 * | 2.23 |
| *bc1810* | enterotoxin | 1.24 * | 1.89 |
| *bc1828* | xaa-pro aminopeptidase, putative | 2.55 | 2.02 |
| *bc1912* | Phage protein | 1.49 * | 1.59 |
| *bc1913* | Phage protein | 1.47 * | 1.85 |
| *bc1935* | hypothetical protein | 1.22 * | 0.57 |
| *bc1952* | hypothetical protein | 0.90 * | 1.59 |
| *bc1978* | siderophore biosynthesis protein, putative | 1.15 * | 2.17 |
| *bc1980* | acyl-CoA synthase | 1.16 * | 1.85 |
| *bc1981* | acyl carrier protein | 1.32 * | 2.12 |
| *bc1983* | hypothetical protein | 1.07 * | 1.67 |
| *bc1992* | hypothetical protein | 0.84 * | 1.61 |
| *bc1993* | mutT/nudix family protein | 0.78 * | 1.51 |
| *bc2011* | general stress protein | 2.26 | 1.94 |
| *bc2018* | oxidoreductase, aldo/keto reductase family | 3.27 | 2.23 |
| *bc2056* | hypothetical protein | 1.92 * | 2.50 |
| *bc2101* | formate--tetrahydrofolate ligase | 3.31 | 2.33 |
| *bc2130* | hypothetical protein | NA | 1.88 |
| *bc2148* | Response regulator aspartate phosphatase inhibitor | 1.89 | 1.89 |
| *bc2170* | sodium-dependent transporter, putative | 2.30 | 1.36 * |
| *bc2179* | acetyltransferase, GNAT family | 1.11 * | 1.78 |
| *bc2223* | Gluconokinase | 4.36 | 3.86 |
| *bc2224* | Gluconate permease | 3.12 | 2.79 |
| *bc2225* | 6-phosphogluconate dehydrogenase | 3.38 | 3.38 |
| *bc2286* | 2-methylcitrate dehydratase | 1.02 * | 1.55 |
| *bc2302* | 2,3-dihydroxybenzoate-2,3-dehydrogenase | 2.43 | 1.73 |
| *bc2303* | isochorismate synthase | 3.90 | 2.31 |
| *bc2304* | 2,3-dihydroxybenzoate-AMP ligase | 5.41 | 2.11 |
| *bc2305* | isochorismatase | 6.64 | 3.43 |
| *bc2306* | Glycine-AMP ligase | 7.12 | 3.22 |
| *bc2307* | nonribosomal peptide synthetase DhbF | 5.29 | 2.25 |
| *bc2308* | Glycine-AMP ligase | 5.59 | 2.58 |
| *bc2310* | drug resistance transporter, EmrB/QacA family | 2.39 | 1.46 |
| *bc2312* | hypothetical protein | 2.43 | 2.12 |
| *bc2390* | hypothetical protein | 3.60 | 3.16 |
| *bc2429* | ABC transporter ATP-binding protein | 1.75 * | 2.24 |
| *bc2436* | SpoIISA like protein | 1.42 * | 1.58 |
| *bc2661* | Bacitracin transport permease protein BCRC | 1.82 * | 1.51 |
| *bc2713* | endo/excinuclease amino terminal domain protein | 0.47 * | 0.63 |
| *bc2740* | preprotein translocase SecY | 1.10 * | 1.65 |
| *bc2750* | hypothetical protein | 1.14 * | 1.57 |
| *bc2757* | tryptophan 2,3-dioxygenase family protein | 2.03 * | 2.11 |
| *bc2758* | hypothetical protein | 2.36 | 1.75 |
| *bc2841* | hypothetical protein | 2.83 | 2.56 |
| *bc2842* | hypothetical protein | 3.22 | 3.17 |
| *bc2896* | aspartate aminotransferase | 2.73 | 1.79 |
| *bc2933* | ABC transporter, ATP-binding protein | 0.49 | 0.69 * |
| *bc2936* | Transcriptional repressor Bm3R1 | 2.00 | 1.69 |
| *bc2939* | prephenate dehydrogenase | 3.42 | 1.39 * |
| *bc2940* | putative aminotransferase | 2.26 | 1.75 |
| *bc2941* | chorismate synthase | 2.20 | 1.58 |
| *bc2942* | 3-deoxy-7-phosphoheptulonate synthase | 6.28 | 4.55 |
| *bc2959* | malate:quinone oxidoreductase | 1.94 * | 2.00 |
| *bc2969* | hypothetical protein | 1.72 * | 1.64 |
| *bc3000* | major facilitator family transporter | 4.53 | 3.58 |
| *bc3024* | nitroreductase family protein | 3.68 | 2.67 |
| *bc3025* | transcriptional regulator, MarR family | 3.81 | 1.93 |
| *bc3054* | hypothetical protein | 1.62 * | 1.56 |
| *bc3093* | Aspartate ammonia-lyase | 0.42 | 0.68 * |
| *bc3094* | L-asparaginase | 0.34 | 0.39 |
| *bc3249* | phosphoserine aminotransferase | 1.25 * | 1.53 |
| *bc3296* | hypothetical protein | 0.35 | 0.57 |
| *bc3355* | CBS domain protein | 2.19 | 1.56 |
| *bc3356* | transcriptional regulator, putative | 1.85 * | 2.49 |
| *bc3380* | alcohol dehydrogenase, zinc-containing | 1.30 * | 1.98 |
| *bc3419* | AMP-binding protein | 1.38 * | 3.12 |
| *bc3462* | hypothetical protein | 1.15 * | 1.79 |
| *bc3463* | arsenical pump family protein | 0.88 * | 1.59 |
| *bc3467* | iron compound ABC transporter, permease protein | 1.45 * | 1.67 |
| *bc3468* | iron compound ABC transporter, permease protein | 1.63 * | 1.76 |
| *bc3539* | cold shock protein CspB | 1.33 * | 1.51 |
| *bc3540* | BNR-repeat containing protein | 2.02 | 2.38 |
| *bc3541* | flavodoxin | 1.95 | 1.22 * |
| *bc3586* | oligopeptide ABC transporter, oligopeptide-binding protein, putative | 1.94 * | 2.39 |
| *bc3600* | serine protease | 1.83 * | 1.65 |
| *bc3605* | hypothetical protein | 1.71 * | 1.55 |
| *bc3616* | aconitate hydratase | 5.60 | 6.13 |
| *bc3640* | Glyoxalase/Bleomycin resistance protein/Dioxygenase superfamily | 0.97 * | 1.69 |
| *bc3650* | imidazolonepropionase | 1.06 * | 1.61 |
| *bc3653* | anti-terminator HutP | 1.72 * | 1.97 |
| *bc3663* | Transporter, Drug/Metabolite Exporter family | 2.23 * | 2.19 |
| *bc3694* | N-acetylmuramoyl-L-alanine amidase | 2.53 | 0.96 * |
| *bc3695* | holin | 1.51 * | 1.53 |
| *bc3706* | transcriptional repressor GlnR | 0.19 | 0.21 |
| *bc3736* | iron compound ABC transporter, permease protein | 1.62 * | 1.57 |
| *bc3743* | peptidase T | 2.01 | 2.10 |
| *bc3774* | pyruvate ferredoxin oxidoreductase, alpha subunit, putative | 1.55 * | 1.64 |
| *bc3777* | phosphoesterase family protein | 0.44 * | 0.57 |
| *bc3784* | IG hypothetical 16623 | 0.95 * | 0.53 |
| *bc3786* | zinc protease, insulinase family | 2.28 | 1.23 * |
| *bc3787* | hypothetical protein | 2.42 | 1.85 |
| *bc3788* | Nucleoside transport system permease protein | 2.21 * | 2.03 |
| *bc3791* | lipoprotein, Bmp family | 1.68 * | 1.68 |
| *bc3792* | transcriptional regulator, GntR family | 2.31 | 1.90 |
| *bc3797* | dihydrodipicolinate synthase | 1.56 * | 1.54 |
| *bc3798* | aspartate kinase I | 1.64 * | 1.82 |
| *bc3805* | polyribonucleotide nucleotidyltransferase | 0.65 * | 0.55 |
| *bc3809* | ribosome-binding factor A | 0.66 * | 0.54 |
| *bc3820* | phosphatidate cytidylyltransferase | 0.52 * | 0.61 |
| *bc3822* | ribosome releasing factor | 0.69 * | 0.54 |
| *bc3823* | uridylate kinase | 0.57 * | 0.52 |
| *bc3824* | elongation factor Ts | 0.57 * | 0.59 |
| *bc3827* | ATP-dependent protease ATP-binding subunit | 0.42 | 0.51 |
| *bc3833* | succinyl-CoA synthetase alpha subunit | 2.19 | 3.70 |
| *bc3834* | succinyl-CoA synthetase subunit beta | 2.35 | 3.55 |
| *bc3844* | hypothetical protein | 0.39 | 0.41 |
| *bc3845* | signal recognition particle-docking protein FtsY | 0.63 * | 0.60 |
| *bc3848* | acyl carrier protein | 0.70 * | 0.61 |
| *bc3849* | 3-ketoacyl-(acyl-carrier-protein) reductase | 0.52 * | 0.44 |
| *bc3850* | acyl-carrier-protein S-malonyltransferase | 0.39 | 0.43 |
| *bc3851* | fatty acid/phospholipid synthesis protein | 0.42 * | 0.39 |
| *bc3852* | transcriptional regulator of fatty acid biosynthesis | 0.65 * | 0.52 |
| *bc3856* | ribosomal protein L28 | 0.51 * | 0.56 |
| *bc3877* | hypothetical protein | NA | 0.60 |
| *bc3881* | Phosphoglycolate phosphatase | 1.50 * | 1.55 |
| *bc3882* | orotate phosphoribosyltransferase | 0.41 | 0.41 |
| *bc3883* | orotidine 5'-phosphate decarboxylase | 0.32 | 0.49 |
| *bc3884* | dihydroorotate dehydrogenase | 0.27 | 0.34 |
| *bc3885* | dihydroorotate dehydrogenase electron transfer subunit | 0.32 | 0.31 |
| *bc3886* | carbamoyl-phosphate synthase large subunit | 0.25 | 0.51 |
| *bc3887* | carbamoyl-phosphate synthase small subunit | 0.29 | 0.31 |
| *bc3888* | dihydroorotase | 0.39 | 0.50 |
| *bc3890* | uracil permease | 0.88 * | 0.61 |
| *bc3910* | N-acetylglucosaminyl transferase | 1.39 * | 0.66 |
| *bc3919* | hypothetical protein | 1.87 | 1.57 |
| *bc3921* | acetyltransferase, GNAT family | 1.24 * | 1.60 |
| *bc3924* | hypothetical protein | 0.43 | 0.56 |
| *bc3936* | hypothetical protein | 1.44 * | 0.61 |
| *bc3947* | pyruvate carboxylase | 1.33 * | 1.89 |
| *bc3950* | hypothetical protein | 1.07 * | 1.65 |
| *bc3951* | PhoH family protein | 0.99 * | 1.59 |
| *bc3952* | hypothetical protein | 1.06 * | 1.85 |
| *bc3956* | GTP-binding protein TypA | 0.45 | 0.65 * |
| *bc3965* | hypothetical protein | 0.94 * | 1.59 |
| *bc3966* | hypothetical protein | 0.91 * | 1.77 |
| *bc3990* | short chain dehydrogenase | 1.82 * | 2.32 |
| *bc4000* | drug resistance transporter, EmrB/QacA family | 6.07 | 3.97 |
| *bc4003* | 5-methyltetrahydropteroyltriglutamate-- homocysteine methyltransferase | 7.47 | 6.51 |
| *bc4023* | acetyl-CoA acetyltransferase | 1.04 * | 1.55 |
| *bc4045* | nitroreductase family protein | 1.32 * | 1.67 |
| *bc4050* | PTS system, glucose-specific IIABC component | 0.44 | 0.88 * |
| *bc4055* | N-acetylglucosamine-6-phosphate deacetylase | 1.89 | 2.29 |
| *bc4086* | purine nucleoside phosphorylase | 1.69 * | 2.00 |
| *bc4110* | riboflavin synthase subunit alpha | 1.39 * | 2.01 |
| *bc4111* | bifunctional 3,4-dihydroxy-2-butanone 4-phosphate synthase/GTP cyclohydrolase II protein | 1.62 * | 1.82 |
| *bc4112* | riboflavin synthase subunit beta | 1.78 * | 1.59 |
| *bc4151* | hypothetical protein | 1.66 * | 1.53 |
| *bc4156* | hypothetical protein | 1.68 * | 1.92 |
| *bc4157* | dihydrolipoamide acetyltransferase | 4.71 | 5.06 |
| *bc4158* | 3-methyl-2-oxobutanoate dehydrogenase, beta subunit | 5.79 | 7.91 |
| *bc4159* | 3-methyl-2-oxobutanoate dehydrogenase, alpha subunit | 6.43 | 10.03 |
| *bc4160* | dihydrolipoamide dehydrogenase | 5.21 | 6.96 |
| *bc4161* | butyrate kinase | 4.18 | 5.24 |
| *bc4162* | leucine dehydrogenase | 6.33 | 8.90 |
| *bc4163* | phosphate acetyltransferase | 3.76 | 4.43 |
| *bc4168* | hypothetical protein | 0.40 | 0.58 |
| *bc4182* | hypothetical protein | 0.57 * | 0.60 |
| *bc4183* | acetyl-CoA carboxylase | 0.56 * | 0.62 |
| *bc4199* | 3-dehydroquinate dehydratase | 1.66 * | 1.83 |
| *bc4224* | glycine dehydrogenase subunit 2 | 3.55 | 4.00 |
| *bc4225* | glycine dehydrogenase subunit 1 | 4.35 | 5.24 |
| *bc4226* | aminomethyltransferase | 4.33 | 5.79 |
| *bc4272* | superoxide dismutase, Mn | 2.20 | 2.59 |
| *bc4283* | ATP-dependent RNA helicase, DEAD/DEAH box family | 0.71 * | 0.55 |
| *bc4310* | ribosomal protein L11 methyltransferase | 2.08 | 1.61 |
| *bc4311* | chaperone protein dnaJ | 1.82 | 1.72 |
| *bc4312* | molecular chaperone DnaK | 4.82 | 5.77 |
| *bc4313* | GrpE protein | 5.57 | 6.41 |
| *bc4314* | heat-inducible transcription repressor | 5.34 | 3.55 |
| *bc4332* | GTPase family protein | 0.49 | 0.57 |
| *bc4333* | hydrolase, HAD subfamily IIIA | 0.66 * | 0.63 |
| *bc4341* | hypothetical protein | 2.19 | 1.44 * |
| *bc4434* | spo0B-associated GTP-binding protein | 0.36 | 0.41 |
| *bc4435* | sporulation initiation phosphotransferase, putative | 0.38 | 0.39 |
| *bc4469* | delta-aminolevulinic acid dehydratase | 1.72 | 1.56 |
| *bc4375* | uridine kinase | 0.47 * | 0.51 |
| *bc4376* | peptidase, U32 family | 0.62 * | 0.55 |
| *bc4377* | peptidase, U32 family | 1.95 | 0.60 |
| *bc4378* | O-methyltransferase family protein | 0.61 * | 0.66 |
| *bc4402* | adenine phosphoribosyltransferase | 0.54 * | 0.56 |
| *bc4434* | spo0B-associated GTP-binding protein | 0.36 | 0.41 |
| *bc4435* | sporulation initiation phosphotransferase, putative | 0.38 | 0.39 |
| *bc4436* | 50S ribosomal protein L27 | 0.58 * | 0.57 |
| *bc4452* | hypothetical protein | 0.44 * | 0.64 |
| *bc4469* | delta-aminolevulinic acid dehydratase | 1.72 | 1.56 |
| *bc4470* | uroporphyrinogen-III synthetase | 1.50 * | 1.52 |
| *bc4476* | GTP-binding protein | 1.80 * | 2.26 |
| *bc4477* | ATP-dependent protease La 1 | 1.95 | 2.42 |
| *bc4511* | acid phosphatase | 0.54 | 0.66 |
| *bc4516* | succinate dehydrogenase | 1.72 * | 2.84 |
| *bc4517* | succinate dehydrogenase | 2.75 | 3.89 |
| *bc4518* | succinate dehydrogenase, cytochrome b558 subunit | 1.99 * | 2.85 |
| *bc4521* | thioredoxin | 1.81 | 2.52 |
| *bc4523* | electron transfer flavoprotein, beta subunit | 3.64 | 4.21 |
| *bc4524* | enoyl-CoA hydratase | 4.21 | 3.41 |
| *bc4525* | transcriptional regulator, TetR family | 1.57 * | 2.32 |
| *bc4536* | hypothetical protein | 1.24 * | 0.60 |
| *bc4568* | drug resistance transporter, EmrB/QacA family | 20.76 | 5.85 |
| *bc4569* | hypothetical protein | 7.27 | 3.37 |
| *bc4571* | peptidase, M42 family | 2.56 | 2.70 |
| *bc4573* | 50S ribosomal protein L20 | 0.51 * | 0.51 |
| *bc4574* | 50S ribosomal protein L35 | 0.73 * | 0.52 |
| *bc4575* | translation initiation factor IF-3 | 0.55 * | 0.55 |
| *bc4583* | glyceraldehyde-3-phosphate dehydrogenase | 0.97 * | 1.59 |
| *bc4591* | maoC like domain protein | 3.63 | 1.72 |
| *bc4592* | malate dehydrogenase | 5.11 | 6.39 |
| *bc4593* | isocitrate dehydrogenase | 7.43 | 9.57 |
| *bc4594* | citrate synthase | 5.70 | 6.75 |
| *bc4614* | proline dipeptidase | 1.73 | 1.72 * |
| *bc4624* | 3-ketoacyl-(acyl-carrier-protein) reductase | 1.66 * | 1.78 |
| *bc4629* | argininosuccinate lyase | 2.62 * | 2.71 |
| *bc4630* | argininosuccinate synthase | 1.66 * | 1.51 |
| *bc4634* | hypothetical protein | 1.27 * | 1.63 |
| *bc4637* | acetate/propionate kinase | 0.42 | 0.40 |
| *bc4639* | thiol peroxidase | 1.23 * | 1.57 |
| *bc4642* | inorganic polyphosphate/ATP-NAD kinase | 1.41 * | 1.80 |
| *bc4678* | aminopeptidase, putative | 2.23 | 2.56 |
| *bc4693* | peptidase, M42 family | 2.11 | 2.49 |
| *bc4703* | transcriptional regulator, DeoR family | 2.86 | 2.86 |
| *bc4707* | drug resistance transporter, EmrB/QacA family | 2.33 | 0.91 * |
| *bc4714* | transcriptional regulator, CarD family | 2.28 | 1.51 * |
| *bc4719* | molybdopterin converting factor, subunit 2 | 1.91 | 1.39 |
| *bc4721* | Molybdopterin biosynthesis MoeA protein | 1.43 * | 1.61 |
| *bc4762* | phosphoenolpyruvate carboxykinase | 1.87 * | 2.83 |
| *bc4785* | mutT/nudix family protein | 2.79 | 1.96 |
| *bc4792* | cytochrome d ubiquinol oxidase, subunit I | 0.43 * | 0.48 |
| *bc4801* | hypothetical protein | 2.01 | 1.72 |
| *bc4855* | 2-oxoglutarate decarboxylase | 1.72 | 1.32 * |
| *bc4856* | menaquinone-specific isochorismate synthase | 2.29 | 1.66 |
| *bc4870* | L-lactate dehydrogenase | 0.26 * | 0.12 |
| *bc4919* | phosphoglucomutase/phosphomannomutase family protein | 1.57 * | 1.62 |
| *bc4921* | leucyl aminopeptidase | 3.09 | 3.07 |
| *bc4926* | pyridine nucleotide-disulphide oxidoreductase | 1.59 * | 1.83 |
| *bc4935* | hesB/yadR/yfhF family protein | 1.45 * | 1.60 |
| *bc4938* | pyridine nucleotide-disulphide oxidoreductase | 3.03 | 3.12 |
| *bc4952* | nifU domain protein | 1.26 * | 1.66 |
| *bc4961* | hypothetical protein | 1.93 | 1.96 |
| *bc4979* | hypothetical protein | 1.63 * | 2.19 |
| *bc4980* | nifU domain protein | 1.71 * | 1.77 |
| *bc4981* | aminotransferase, class V | 1.98 | 1.95 |
| *bc4982* | hypothetical protein | 1.83 | 1.70 |
| *bc4983* | ABC transporter, ATP-binding protein | 2.00 * | 2.52 |
| *bc4991* | glycine cleavage system protein H | 1.33 * | 1.78 |
| *bc4996* | L-lactate dehydrogenase | 1.17 * | 0.51 |
| *bc5002* | acyl-CoA dehydrogenase | 9.90 | 4.97 |
| *bc5003* | acetyl-CoA acetyltransferase | 13.93 | 5.62 |
| *bc5004* | 3-hydroxyacyl-CoA dehydrogenase/enoyl-CoA hydratase/isomerase family protein | 5.50 | 3.19 |
| *bc5006* | proline dehydrogenase family protein | 4.73 | 3.70 |
| *bc5013* | amino acid permease family protein | 1.50 * | 1.55 |
| *bc5016* | OxaA-like protein precursor | 1.62 * | 2.15 |
| *bc5034* | methyl-accepting chemotaxis protein | 0.44 * | 0.66 |
| *bc5048* | ferritin | 1.52 * | 2.05 |
| *bc5051* | sodium/alanine symporter family protein | 0.19 | 0.42 |
| *bc5060* | hypothetical protein | 1.64 * | 1.69 |
| *bc5061* | pyridine nucleotide-disulphide oxidoreductase | 1.77 * | 2.25 |
| *bc5062* | tyrosyl-tRNA synthetase | 1.96 | 1.41 * |
| *bc5075* | LSU ribosomal protein L11P | 6.10 | 6.67 |
| *bc5076* | Short chain dehydrogenase | 4.08 | 5.36 |
| *bc5077* | hypothetical protein | 3.29 | 5.06 |
| *bc5078* | LSU ribosomal protein L11P | 3.16 | 4.16 |
| *bc5085* | hypothetical Cytosolic Protein | 1.59 * | 1.70 |
| *bc5087* | putative lantibiotic precursor peptide | 6.25 | 6.77 |
| *bc5088* | putative lantibiotic precursor peptide | 9.49 | 7.62 |
| *bc5089* | putative lantibiotic precursor peptide | 4.90 | 6.16 |
| *bc5090* | putative lantibiotic precursor peptide | 5.57 | 5.89 |
| *bc5091* | SnoK-like protein | 1.62 * | 1.67 |
| *bc5092* | Glyoxalase/Bleomycin resistance protein/Dioxygenase superfamily | 1.73 * | 2.30 |
| *bc5095* | Transposase | 1.28 * | 1.63 |
| *bc5096* | hypothetical protein | 0.86 * | 1.51 |
| *bc5104* | iron compound ABC transporter, permease protein | 1.65 * | 1.76 |
| *bc5105* | iron compound ABC transporter, permease protein | 1.58 * | 1.86 |
| *bc5106* | iron compound ABC transporter, iron compound-binding protein | 1.91 * | 2.14 |
| *bc5126* | Transposase | 1.25 * | 1.61 |
| *bc5129* | ribonuclease R | 1.64 * | 1.77 |
| *bc5130* | carboxylesterase | 1.75 | 2.14 |
| *bc5140* | glyceraldehyde-3-phosphate dehydrogenase | 0.48 * | 0.33 |
| *bc5141* | gapA transcriptional regulator CggR | 0.38 * | 0.22 |
| *bc5152* | ATP-dependent Clp protease proteolytic subunit | 6.92 | 6.47 |
| *bc5157* | mutT/nudix family protein | 1.58 * | 1.93 |
| *bc5159* | thioredoxin reductase | 1.95 | 1.95 |
| *bc5162* | hydrolase, haloacid dehalogenase-like family | 1.73 * | 1.52 |
| *bc5163* | prolipoprotein diacylglyceryl transferase | 1.94 * | 1.69 |
| *bc5164* | HPr kinase/phosphorylase | 2.08 | 1.77 |
| *bc5184* | carboxyl-terminal protease | 1.36 * | 1.61 |
| *bc5186* | cell division ABC transporter, ATP-binding protein FtsE | 0.50 | 0.78 * |
| *bc5190* | ribosomal subunit interface protein | 2.66 | 3.65 |
| *bc5191* | cold shock protein CspC | 0.30 | 0.51 |
| *bc5192* | comF operon protein 3 | 0.36 | 0.65 |
| *bc5199* | conserved hypothetical protein TIGR00257 | 1.34 * | 1.53 * |
| *bc5203* | glycosyl transferase, group 4 family protein | 1.92 | 1.65 |
| *bc5232* | sulfatase | 1.91 | 1.71 |
| *bc5239* | hypothetical protein | 0.48 | 0.63 |
| *bc5243* | hypothetical protein | 0.35 | 0.65 |
| *bc5266* | Heteropolysaccharide repeat unit export protein | 0.58 * | 0.58 |
| *bc5271* | UDP-N-acetylglucosamine 4-epimerase | 0.47 | 0.66 * |
| *bc5272* | Carbamoyl-phosphate synthase small chain | 0.48 | 0.54 |
| *bc5273* | UDP-bacillosamine synthetase | 0.45 | 0.55 |
| *bc5277* | Tyrosine-protein kinase (capsular polysaccharide biosynthesis) | 0.46 | 0.56 |
| *bc5279* | Tyrosine-protein kinase (capsular polysaccharide biosynthesis) | 2.29 | 2.73 |
| *bc5280* | (3R)-hydroxymyristoyl ACP dehydratase | 1.52 * | 1.77 |
| *bc5288* | UDP-N-acetylglucosamine 1-carboxyvinyltransferase | 0.66 * | 0.60 |
| *bc5311* | ATP synthase subunit C | 1.18 * | 1.62 |
| *bc5315* | uracil phosphoribosyltransferase | 0.55 * | 0.56 |
| *bc5320* | PTS system, glucose-specific IIA component, putative | 1.70 * | 1.55 |
| *bc5322* | hypothetical protein | 3.56 | 3.08 |
| *bc5330* | thymidine kinase | 0.32 | 0.42 |
| *bc5331* | 50S ribosomal protein L31 | 0.42 | 0.48 |
| *bc5333* | fructose-1,6-bisphosphatase, class II | 1.71 * | 1.82 |
| *bc5334* | UDP-N-acetylglucosamine 1-carboxyvinyltransferase | 1.64 * | 2.20 |
| *bc5335* | fructose-bisphosphate aldolase | 2.09 | 2.34 |
| *bc5336* | stage 0 sporulation protein F | 1.79 * | 1.68 |
| *bc5338* | CTP synthetase | 0.45 * | 0.49 |
| *bc5351* | Bacillolysin | 1.45 * | 2.50 |
| *bc5365* | hypothetical protein | 0.52 * | 0.60 |
| *bc5372* | multidrug resistance protein, putative | 2.86 | 1.87 |
| *bc5377* | hypothetical protein | 2.13 | 1.51 |
| *bc5379* | HD domain protein | 1.96 | 1.55 |
| *bc5388* | hypothetical protein | 1.51 * | 1.52 |
| *bc5392* | hypothetical protein | 1.78 * | 1.65 |
| *bc5398* | uracil-DNA glycosylase | 1.65 * | 2.21 |
| *bc5399* | ABC transporter, permease protein | 4.25 | 1.93 |
| *bc5401* | lipase/acylhydrolase, putative | 2.42 | 1.51 |
| *bc5410* | acyl carrier protein phosphodiesterase | 0.39 | 0.59 |
| *bc5413* | phosphomethylpyrimidine kinase | 2.24 | 2.23 |
| *bc5426* | cytosolic long-chain acyl-CoA thioester hydrolase family protein | 1.80 * | 1.63 |
| *bc5435* | transporter, AcrB/AcrD/AcrF family | 2.07 | 2.43 |
| *bc5437* | hypothetical protein | 1.70 * | 1.52 |
| *bc5453* | oligoendopeptidase F, putative | 1.47 * | 1.68 |
| *bc5460* | yycI protein | 1.40 * | 1.51 |
| *bc5462* | sensory box histidine kinase YycG | 1.78 | 1.52 |
| *bc5463* | DNA-binding response regulator YycF | 1.50 * | 1.86 |
| *bc5481* | stage 0 sporulation protein J | 1.56 * | 1.53 |
|  |  |  |  |
